# A Comprehensive Understanding of Post-Translational Modification of Sox2 via Acetylation and *O*-GlcNAcylation in Colorectal Cancer

**DOI:** 10.3390/cancers16051035

**Published:** 2024-03-03

**Authors:** Yoojeong Seo, Dong Keon Kim, Jihye Park, Soo Jung Park, Jae Jun Park, Jae Hee Cheon, Tae Il Kim

**Affiliations:** 1 Division of Gastroenterology, Department of Internal Medicine, Institute of Gastroenterology, Severance Hospital, Yonsei University College of Medicine, Seoul 03722, Republic of Korea; yjseo90@yuhs.ac (Y.S.); kimdk0514@yuhs.ac (D.K.K.); wisdompark@yuhs.ac (J.P.); sjpark@yuhs.ac (S.J.P.); jaejpark@yuhs.ac (J.J.P.); geniushee@yuhs.ac (J.H.C.); 2 Graduate School of Medical Science, Brain Korea 21 Project, Yonsei University College of Medicine, Seoul 03722, Republic of Korea; 3 Yonsei Cancer Prevention Center, Severance Hospital, Yonsei University College of Medicine, Seoul 03722, Republic of Korea

**Keywords:** Sox2, Colorectal Cancer (CRC), post-translational modification (PTM), acetylation, histone deacetylase (HDAC), *O*-GlcNAcylation

## Abstract

**Simple Summary:**

This study provides insights into the regulation of Sox2, specifically focusing on the acetylation of Sox2 K75 residue and *O*-GlcNAcylation of S246 residue. These post-translational modifications (PTM) are orchestrated through interactions with ACSS2/p300 for acetylation and miR29a/HDAC4 for deacetylation, respectively. These findings shed light on the significance of these PTMs in the development and progression of colorectal cancer.

**Abstract:**

Aberrant expression of the pluripotency-associated transcription factor Sox2 is associated with poor prognosis in colorectal cancer (CRC). We investigated the regulatory roles of major post-translational modifications in Sox2 using two CRC cell lines, SW480 and SW620, derived from the same patient but with low and high Sox2 expression, respectively. Acetylation of K75 in the Sox2 nuclear export signal was relatively increased in SW480 cells and promotes Sox2 nucleocytoplasmic shuttling and proteasomal degradation of Sox2. LC-MS-based proteomics analysis identified HDAC4 and p300 as binding partners involved in the acetylation-mediated control of Sox2 expression in the nucleus. Sox2 K75 acetylation is mediated by the acetyltransferase activity of CBP/p300 and ACSS3. In SW620 cells, HDAC4 deacetylates K75 and is regulated by miR29a. *O*-GlcNAcylation on S246, in addition to K75 acetylation, also regulates Sox2 stability. These findings provide insights into the regulation of Sox2 through multiple post-translational modifications and pathways in CRC.

## 1. Introduction

Colorectal cancer (CRC) ranks as the third leading cause of cancer-related deaths worldwide [1], with a significant number of patients diagnosed at advanced stages characterized by treatment resistance, recurrence, and metastasis [2,3]. Sox2, a member of the SRY-related HMG-box family, is a transcription factor known for its role in mammalian embryogenesis and stem cell maintenance [4]. Sox2 has emerged as a crucial factor in cancer progression, showing aberrant expression in various human cancers, including CRC [5]. In addition, high Sox2 expression has been associated with poor prognosis and resistance to chemotherapy and radiotherapy, potentially as a result of its influence on cancer stem cell characteristics [6]. The underlying mechanisms driving Sox2 upregulation in the oncogenic process remain unclear [7].

Post-translational modifications (PTMs) such as acetylation, *O*-GlcNAcylation, methylation, phosphorylation, and SUMOylation play critical roles in regulating the transcriptional activity of Sox2 by changing its nuclear localization and stability (Supplementary Figure S1). Most of these modifications affect the nucleocytoplasmic shuttling of Sox2, possibly by modulating interactions with binding partners and cofactors [8]. The precise impact of each PTM on Sox2 activity in CRC remains elusive, however, and it is essential to meticulously identify and characterize these modifications to determine their functional significance in CRC.

Sox2 includes a nuclear export signal (NES), where acetylation of the K75 residue by CREB-binding protein (CBP)/p300 transcriptional coactivating protein promotes nuclear export of Sox2 and subsequent proteasomal degradation in embryonic stem cells. However, there have been no reports on the significance of K75 acetylation of Sox2 in cancers [9]. Furthermore, despite the known involvement of histone deacetylases (HDACs) in cancer development and progression, the potential relationship between Sox2 and HDACs, particularly HDAC4, in CRC has received little study. Another potentially important PTM, *O*-GlcNAcylation, has been found to be increased in CRC, facilitating the proliferative and migratory properties of tumor cells and promoting CRC metastasis [10,11]. *O*-GlcNAcylation occurs at two positions in Sox2: S248 and T258 [12]. *O*-GlcNAcylation in the S246 region can impact Sox2 transcriptional activity and regulate cellular self-renewal in pancreatic cancer [13]; however, the role of Sox2 *O*-GlcNAcylation in CRC has received little study. In addition, the mechanisms of Sox2 PTMs and their comprehensive interactions in CRC remain poorly understood.

We investigated the major PTMs of Sox2, their interactive regulators, and their roles in the regulation of Sox2 expression. We found that acetylation of K75 mediated by acetyl-CoA synthetase 2 (ACSS2)/p300, along with deacetylation mediated by the miR29a-HDAC4 axis and *O*-GlcNAcylation of S246, are involved in regulating the stability of Sox2 during CRC progression.

## 2. Materials and Methods

### 2.1. Cell Lines and Culture Conditions

CRC cell lines were purchased from the American Type Culture Collection (Manassas, VA, USA). All cell lines were maintained in Dulbecco’s modified Eagle’s medium (DMEM; Invitrogen, Carlsbad, CA, USA) supplemented with 10% fetal bovine serum (Gibco, Franklin Lakes, NJ, USA) and 1% penicillin/streptomycin (Invitrogen, Carlsbad, CA, USA) at 37 °C in 5% CO_2._

### 2.2. Drugs

Trichostatin A (TSA), 5′-azacytidine (5-AZA), proteasome inhibitor MG-132, histone acetyltransferase p300/CBP inhibitor C646, and inhibitor of *O*-GlcNAcase Thiamet-G were purchased from Sigma-Aldrich (St. Louis, MO, USA). VY-3-135, a potent ACSS2 inhibitor, was purchased from Selleckchem (Houston, TX, USA)

### 2.3. RNAi

For the RNAi experiments, siRNAs targeting Sox2, HDAC4, and HDAC1 were purchased from Dharmacon (Lafayette, CO, USA). SMARTpool mixes of ON-TARGETplus siRNAs (GE Healthcare, Lafayette, CO, USA) were used as non-targeting controls according to the manufacturer’s instructions. Dr. Jinwoo Lee (Yonsei University) kindly provided siRNAs targeting OGA and OGT. Pools of siRNAs were transfected using INTERFEin (Polyplus, New York, NY, USA) as indicated with a final siRNA concentration of 20 nM. Cells were transfected twice, first by reverse transfection and again 24 h later by forward transfection as outlined in the manufacturer’s instructions.

### 2.4. DNA and microRNA Transfection

For Sox2 overexpression in SW480 cells, we treated the cells with pcDNA-Sox2 kindly provided by Dr. Jinwoo Lee (Yonsei University). We subcloned pcDNA-MYC-Sox2 wild-type and pcDNA-MYC-Sox2-K75A (acetylation prevention), -K75R (acetylation prevention), -K75Q (acetylation mimic), -S246A (*O*-GlcNAcylation null), -S246D (no effect on *O*-GlcNAcylation), and -K75Q/S246A (acetylation mimic/*O*-GlcNAc prevention) mutant vectors and performed transient transfection with jetPRIME (Polyplus, New York, NY, USA). For doxycycline-inducible Sox2 overexpression in SW480 cells, we subcloned a pCW57-FLAG-Sox2 wild-type puromycin selection vector. For OGT and OGA overexpression in SW480 and SW620 cells, we treated the cells with pCMV-Flag-OGA and pCMV-Flag-OGT mammalian expression vectors kindly provided by Dr. Jin Won Cho (Yonsei University). We purchased microRNA-29a mimics from Addgene (Watertown, MA, USA) and Dharmacon (GE Healthcare, Lafayette, CO, USA).

### 2.5. Transfection and Lentiviral Transduction

Cells were transfected with constructs using jetPRIME (Polyplus, New York, NY, USA) for 48 h according to the manufacturer’s instructions. For preparation of virus particles, HEK 293T cells were transfected with plasmids encoding pMD3.G and psPAX2 together with constructs cloned into the lentiviral vector. Virus-containing supernatants were obtained 48 h post-transfection and filtered through a 0.45 μm filter. The cells were transduced with virus by centrifugation at 2000 rpm for 30 min and then incubated for 4 h. Successfully transduced cells were selected with puromycin.

### 2.6. Reverse-Transcription Quantitative Real-Time Polymerase Chain Reaction (RT-qPCR) for miRNA Quantitation

We synthesized cDNA using the miRCURY LNA miRNA PCR Starter Kit (Quiagen, Germantown, MD, USA) per the manufacturer’s instructions. One first-strand cDNA synthesis reaction per sample was performed. Two hundred nanograms of total RNA per 10 μL cDNA synthesis reaction was used as per the manufacturer’s instructions for all samples except for plasma samples, for which 10 ng total RNA per 10 μL cDNA synthesis reaction was used.

### 2.7. Western Blot Analysis

Cells were lysed and sonicated in NETN (0.5% Nonidet P-40, 20 mM Tris [pH 8.0], 50 mM NaCl, 50 mM NaF, 100 µM Na_3_VO_4_, 1mM DTT, and 50 µg/mL PMSF) at 4 °C. Crude lysates were cleared by centrifugation at 14,000 rpm at 4 °C for 5 min. After protein quantification, 40 μg of the protein extracts were fractionated using 10% or 12% sodium dodecyl sulfate–polyacrylamide gel electrophoresis (SDS-PAGE) and then transferred to a polyvinylidene fluoride membrane (Bio-Rad, Hercules, CA, USA). After blocking with 5% skim milk, the membrane was incubated with primary antibodies overnight at 4 °C. Subsequently, the membrane was incubated with secondary antibodies for 1 h at room temperature. To express the light emission of the proteins, an Enhanced Chemiluminescence Western blot detection kit (Amersham Biosciences, Freiburg, Germany) was used, and the expressed light was captured on Kodak image film. All antibody information is summarized in Supplementary Table S1.

### 2.8. RNA Extraction and qPCR

Total RNA was isolated using TRIZOL reagent (Invitrogen, Carlsbad, CA, USA). Equal amounts of cDNA were synthesized using the reverse transcription 5× Master Pro Mix (ELPISBIO, Daejeon, Republic of Korea) and mixed with 2× SYBR Green with high ROX (ELPISBIO, Daejeon, Republic of Korea). Then, qPCR was performed using gene-specific primers. The qPCR cycling conditions were: 55 °C for 10 min, 95 °C for 10 min, followed by 40 cycles of 95 °C for 15 s and 60 °C for 1 min. The specificity of the reaction was verified by melting curve analysis.

### 2.9. Immunohistochemistry of Patient CRC Tissues

We obtained 165 stage III CRC samples by surgical resection from patients and an additional 21 metastatic recurrence samples from the same patients at Severance Hospital of the Yonsei University (Seoul, Republic of Korea) from January 2011 to December 2012 (IRB#4-2012-0859). The dissected tumor samples were stored as 4 μm sections of formalin-fixed, paraffin-embedded tissue. The paraffin-embedded sections were deparaffinized in xylene and rehydrated in gradually decreasing concentrations of ethanol. Antigen retrieval was performed using sodium citrate buffer (10 mM, pH 6.0) in a heated pressure cooker for 5 or 7 min. After incubation with 3% hydrogen peroxide for 30 min to block endogenous peroxidase activity, the sections were incubated in a blocking reagent for 30 min at room temperature. The sections were then incubated with the primary antibody overnight at 4 °C, followed by the secondary antibody for 30 min at room temperature. After the slides were developed with a Vectastain ABC kit (Vector Laboratories, Burlingame, CA, USA), immunodetection was performed using DAB solution (Dako, Carpinteria, CA, USA). After counterstaining with hematoxylin, immunohistochemical staining was evaluated by light microscopy, and immunoactivity was assessed based on the proportion of immunostained tumor cells.

### 2.10. Immunofluorescence and Confocal Imaging

For immunofluorescence and confocal imaging, cells were cultured in a confocal dish, fixed with 4% paraformaldehyde at 4 °C for 15 min, and then permeabilized with 1% Triton X-100 (Sigma-Aldrich, St. Louis, MO, USA)/1% BSA at room temperature for 10 min. After being washed twice with PBS, the cells were blocked with 1% BSA at room temperature for 1 h and then incubated with primary antibodies at 4 °C overnight. After another washing, fluorescence-conjugated secondary antibodies were added to the cells and incubated at room temperature for 1 h. The cells were then washed again and DAPI was added, followed by another wash. Cells were analyzed with an LSM700 laser scanning confocal microscope (Carl Zeiss, Oberkochen, Germany).

### 2.11. Immunoprecipitation

For immunoprecipitation, cells were washed with ice-cold PBS and then lysed in NETN buffer at 4 °C for 10 min. Crude lysates were cleared by centrifugation at 14,000 rpm at 4 °C for 5 min. The resulting supernatants were incubated with proteinA-agarose-conjugated primary antibodies. The immunocomplexes were then washed thrice with NETN buffer and subjected to SDS-PAGE and Western blotting.

### 2.12. Cell Fractionation

For cytosolic and nuclear protein fractionation, cells were lysed in cytosol extraction buffer [10 mM HEPES (pH 7.9), 10 mM KCl, 0.1 mM EDTA, 0.1 mM EGTA, 1 mM DTT, and 0.5% NP-40] on ice for 15 min and then centrifuged at 4000 rpm for 10 min. After the supernatants were collected as the cytosolic extract, the pellets were lysed in hypertonic buffer [20 mM HEPES (pH 7.9) 0.4 M NaCl, 1 mM EDTA, 1 mM EGTA, and 1 mM DTT] on ice for 20 min. The lysed samples were centrifuged at 14,000 rpm for 20 min, and the supernatant was collected as the nuclear extract.

### 2.13. Expression Profile Datasets

The public dataset GSE39582 was obtained from the National Center for Biotechnology Information (NCBI) Gene Expression Omnibus (GEO) database. Expression profiles were analyzed using GSEA version 2.1.0 software.

### 2.14. Gene Expression Analysis for mRNA Array and microRNA Array

Microarray experiments were conducted using HumanHT-12 v4 Sentrix Expression BeadChip (Illumina, San Diego, CA, USA). Hybridization of labeled-cRNA to the BeadChip, washing, and scanning were performed according to the Illumina Bead Station 500G manual. Extraction of mRNA expression data and statistical analysis of raw data were performed using software provided by the manufacturer (Illumina GenomeStudio v2011.1). Expression intensities were normalized by quantile normalization. Using the normalized intensities, genes with differential expression between SW480 cells and SW620 cells were determined by a previously reported integrated statistical method [14].

### 2.15. Immunoprecipitation for Mass Spectrometric Analysis

Sox2-binding proteins were immunoprecipitated using anti-Flag antibody-conjugated agarose beads (80 μL of 50% slurry) from about 90 mg of extracts that had been washed with buffer [20 mM Tris-HCl (pH 7.9), 15% glycerol, 1 mM EDTA, 1 mM dithiothreitol (DTT), 0.2 mM PMSF, 0.05% Nonidet P40, and 150 mM KCl] to remove non-specific contaminants. The bound materials were eluted by competition with the Flag peptide (0.1 mg/mL), resolved by SDS-PAGE, and then stained with Coomassie blue for LC-MS/MS analysis.

### 2.16. In-Gel Digestion

Flag-Sox2 or endogenous Sox2 bands were excised from SDS-PAGE gels and destained with washing solution [25 mM NH4HC03 with 50% acetonitrile (ACN)]. After dehydration in 100% ACN, the gel pieces were dried in a SpeedVac. The samples were then incubated with trypsin (0.025 µg/µL) or chymotrypsin (0.025 µg/µL) for 18 h at 37 °C. The digested peptides were extracted with 50% ACN twice and dried in a SpeedVac.

### 2.17. LC-MS/MS Analysis

LC-MS/MS analyses to identify Sox2 binding partners and PTMs were performed at the Yonsei Proteome Research Center and Proteome Tech Inc. (Seoul, Republic of Korea), respectively.

### 2.18. LC-MS/MS Data Analysis for PTMs

All raw files were converted to mgf files using Proteowizard [15]. Data files were searched with X!tandem [16], MyriMatch [17], and MS-GF+ [18] using SearchGUI [19] against the *Homo sapiens* database on UniProt and using PeptideShaker [20] against a custom database downloaded from UniProt. Search parameters were set as follows: carbamidomethylation of cysteine (+57.0125) as a fixed modification; oxidation of methionine (+15.9949), acetylation of protein N-terminus (+43.0106), and acetylation of lysine (+ 43.0106) as variable modifications; precursor and fragments mass tolerance set to 10 ppm; 0.5 Da mass tolerance for precursor and product ions; fully tryptic digestion with up to two missed cleavages.

### 2.19. Survival Analysis

For all survival analyses, Kaplan–Meier survival plots were generated, and statistical significance was determined by the log-rank test. Hazard ratios and 95% confidence intervals were determined by univariate or multivariate Cox proportional hazards regression to identify the impact of HDAC4 levels on survival probability.

### 2.20. RNA-seq and Bioinformatics Analysis of Patient-Derived Organoids

All samples used for organoid bulk RNA-seq and biological analyses were obtained from Severance Hospital of Yonsei University (IRB# 4-2012-0859). This study was approved by the Ethics Committees of both institutes. Healthy and neoplastic colonic tissues were obtained from either endoscopic biopsy samples or surgically resected specimens. Total RNA extraction, RNA library preparation, and RNA-seq and bioinformatics analyses were performed as described previously [21] with some modifications. RNA-seq was performed using the Nova-Seq 6000 sequencing system (Illumina) by Macrogen. Differentially expressed genes were filtered based on a fold change  >  3. Genes with transcripts per million values < 10 in all samples were excluded from the analysis. Differentially expressed genes were subjected to core analysis using Ingenuity Pathway Analysis software (Qiagen, Germantown, MD, USA, version 42012434). The identified differentially expressed genes were subjected to subanalyses of diseases and functions, canonical pathways, and upstream regulators.

### 2.21. Statistical Analysis

Statistical analyses were performed using IBM SPSS Statistics version 20.0 (IBM Co., Armonk, NY, USA). For the evaluation of two groups, unpaired Student’s *t*-tests or Mann–Whitney tests were performed. For the evaluation of more than two groups, one-way or two-way ANOVA was applied. Every experiment was conducted at least in triplicate to ensure reliability. All calculated *p*-values were two sided, and *p*  <  0.05 was considered statistically significant.

## 3. Results

### 3.1. K75-Lysine Acetylation Induces Proteasomal Degradation of Sox2

**Figure 1:**
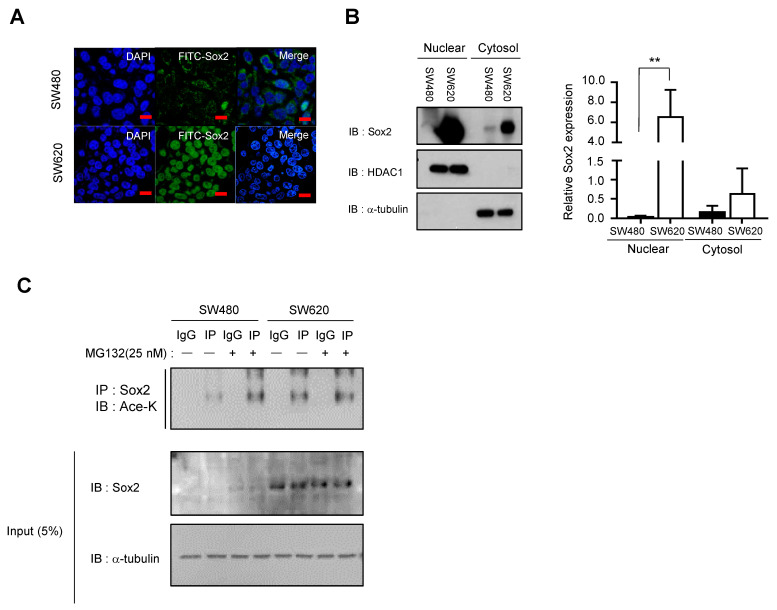
Difference of Sox2 expression and lysine acetylation between SW480 cells and SW620 cells. (**A**) Immunofluorescence staining of SW480 and SW620 cells with anti-Sox2 antibody (green) reveals differential expression of endogenous Sox2 between the two cell lines. Scale bar, 10 μm. (**B**) Nuclear and cytosolic fractions were obtained by fractionation followed by Western blot analysis to examine the amount and localization of Sox2 in SW480 and SW620 cells. HDAC1 and α-tubulin were used as loading and fractionation controls for the nuclear and cytosolic fractions, respectively. Data represent three independent experiments. Bar graphs depict the means ± SEM. Statistical significance was determined using unpaired Student’s *t*-tests (** *p* < 0.005). Full-length blots/gels are presented in Supplementary Figure S7. (**C**) Immunoprecipitation using anti-Sox2 and immunoblotting using Ace-K antibody were performed after SW480 and SW620 cells were treated with MG132 (25 nM) for 24 h. Full-length blots/gels are presented in Supplementary Figure S7.

Sox2 expression is rare in normal colon tissue; however, it significantly increases during CRC tumorigenesis [22]. We observed progressive increases in Sox2 expression as tumorigenesis advanced in tumors of APC^min/+^ mice and human normal colon, primary CRC, and CRC metastasis tissues (Supplementary Figure S2A). Furthermore, using public data on patients with CRC, we found that patients with high Sox2 expression have a poorer prognosis than those with relatively low Sox2 expression (Supplementary Figure S2B). In light of these findings, we performed in vitro experiments with two CRC cell lines, SW480 and SW620, derived from a primary colon adenocarcinoma and metastatic lymph node of the same patient, respectively. Despite sharing common genetic backgrounds, SW620 cells displayed higher expression of Sox2, particularly in the nucleus, compared with SW480 cells (Figure 1A,B).

**Figure 2:**
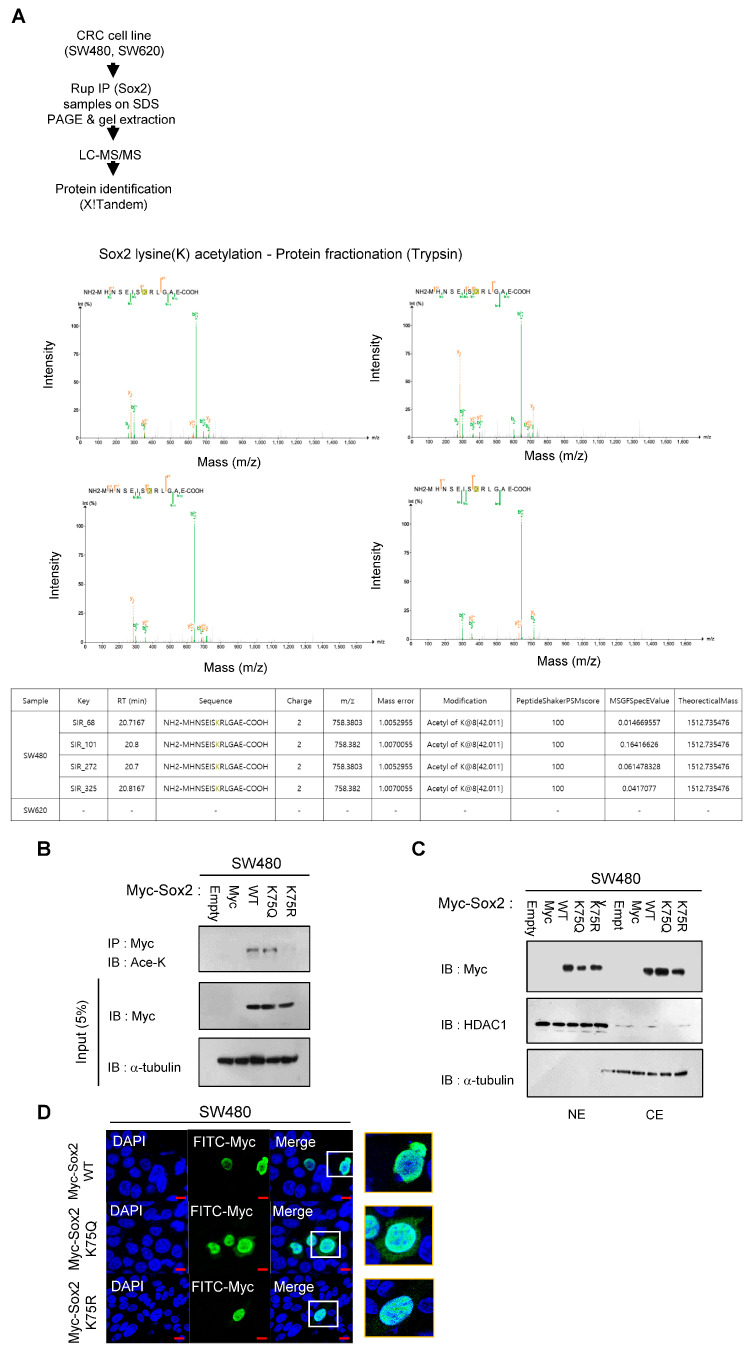
Difference in K75 acetylation between SW480 cells and SW620 cells. (**A**) Acetylation of the K75 residue in SW480 and SW620 cells was verified by manual inspection of LC-MS/MS spectra. Endogenous Sox2 proteins from SW480 and SW620 cells were fractionated with trypsin, and the difference in K75 acetylation within the ‘MHNSEISKRLGAE’ sequence was confirmed. Details of the mass spectrometric analysis and database search can be found in the Materials and Methods section. (**B**) SW480 cells were transfected with plasmids encoding Myc-Sox2 wild-type and K75Q and K75R mutants. After 48 h, the transfected cells were subjected to immunoprecipitation using an anti-Myc antibody. Western blot was subsequently performed using anti-Ace-K, Myc, and a-tubulin antibodies. Full-length blots/gels are presented in Supplementary Figure S7. (**C**) The subcellular localization of Sox2 was determined in nuclear (NE) and cytosolic (CE) fractions of SW480 cells transfected with Myc-Sox2 wild-type and K75Q and K75R mutants. HDAC1 and α-tubulin were used as loading and fractionation controls. Full-length blots/gels are presented in Supplementary Figure S7. (**D**) SW480 cells were transfected with Myc-Sox2 wild-type and K75Q and K75R mutants for 48 h and then fixed and stained with anti-Myc (green). DAPI was used to indicate the nuclei. Scale bar, 10 μm.

Given that acetylation is a major PTM of Sox2, we conducted an immunoprecipitation analysis to compare the levels of Sox2 acetylation between the SW480 and SW620 cell lines. Our observations revealed that SW480 cells had higher levels of Sox2 lysine acetylation compared with SW620 cells. Furthermore, treatment with the proteasome degradation inhibitor MG132 resulted in increased overall Sox2 protein levels in SW480 cells, suggesting that proteasomal degradation of Sox2 might be mediated by lysine acetylation (Figure 1C). Additionally, our PTM analysis (Figure 2A) demonstrated that compared with SW620 cells, SW480 cells had elevated acetylation at the Sox2 K75 residue, which is associated with the Sox2 NES. To further investigate the role of K75 acetylation in the NES of Sox2 in SW480 cells, we conducted transfections using Sox2 wild-type and K75 mutant constructs. We found that an acetylation-mimic mutant (K75Q) exhibited higher Sox2 acetylation levels, resulting in increased cytosolic expression of Sox2, whereas an acetylation-prevention mutant (K75R) showed decreased acetylation and higher nuclear expression of Sox2 (Figure 2B–D).

### 3.2. CBP/p300-Mediated Acetylation Contributes to Nuclear Export and Degradation of Sox2

**Figure 3:**
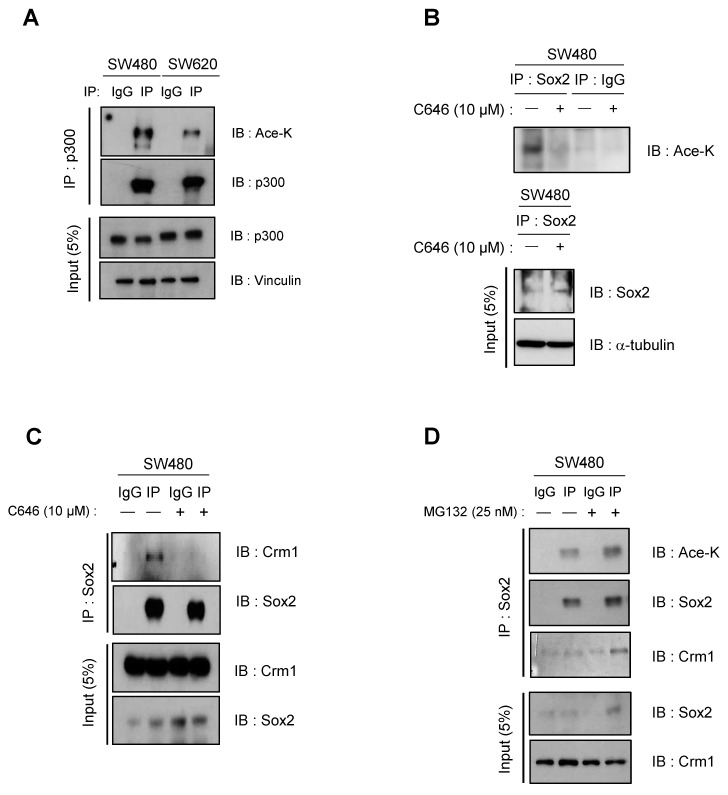
P300-induced lysine acetylation of Sox2 induces nuclear export of Sox2. (**A**) Immunoprecipitation was performed using anti-p300 antibody on SW480 and SW620 cell lysates, followed by immunoblotting using antibodies against Acetyl-lysine, p300, and vinculin. Full-length blots/gels are presented in Supplementary Figure S7. (**B**,**C**) Immunoprecipitation of Sox2 was performed on SW480 cell lysates treated with or without the histone acetyltransferase p300 inhibitor C646 (10 μM) for 24 h. Western blot was performed using antibodies against Acetyl-lysine, Sox2 a-tubulin and Crm1. Full-length blots/gels are presented in Supplementary Figure S7. (**D**) To assess Sox2 binding to Crm1 under conditions of protection from proteasomal degradation, SW480 cells were treated with MG132 (25 nM) for 24 h. Immunoprecipitation was then performed using anti-Sox2, followed by immunoblotting with an Ace-K antibody and anti-Crm1. Full-length blots/gels are presented in Supplementary Figure S7.

CBP/p300 was reported to promote the transcriptional activation of Sox2 by regulating its acetylation in lung cancer [23]. In addition, acetylation at K1499 was shown to enhance the histone acetyltransferase activity of p300 and impact various signaling events [24]. We quantified the levels of acetylated lysine residues in p300 and confirmed that they were higher in SW480 cells than in SW620 cells (Figure 3A). The nuclear export of proteins containing NESs is regulated by interactions with the nuclear export receptor protein Crm1, which is known to mediate the nuclear export of Sox2 [9]. We observed that reducing p300 activity by treating with the histone acetyltransferase inhibitor C646 resulted in decreased acetylation of Sox2 and increased expression of Sox2, consequently disrupting the interaction between Sox2 and Crm1 in SW480 cells (Figure 3B,C). Conversely, treatment of SW480 cells with MG132 resulted in increased lysine acetylation in Sox2 and restored the interaction between Crm1 and Sox2 (Figure 3D). These findings suggest that p300-induced lysine acetylation of Sox2 contributes to Sox2 nuclear export and subsequent proteasomal degradation.

### 3.3. ACSS2-Mediated Modulation of p300/CBP Activity Regulates Sox2 Expression and Stability

To investigate the changes that occur when Sox2 expression is increased in SW480 cells, we generated a stable cell line derived from SW480 cells that expresses Flag-tagged wild-type Sox2 from a doxycycline-inducible vector. Sox2 protein expression in the stable cell line increased within 48 h following doxycycline induction; however, the nuclear expression of Sox2 was not sustained and exhibited a relative decrease after 72 h (Supplementary Figure S3A,B). To comprehensively understand the acetylation and binding proteins associated with Sox2, we examined the Sox2 degradation process using Flag-immunoprecipitation and gel extraction followed by LC-MS/MS analysis of Flag-Sox2-expressing SW480 cells treated for 48 h or 72 h with doxycycline to increase Sox2 expression. To optimize suppression of Sox2 degradation, we also treated Flag-Sox2-expressing SW480 cells for 4, 8, 12, or 24 h with MG132 after 48 h of doxycycline treatment (Supplementary Figure S3C). For the acetylation and binding partner analysis, we performed LC-MS/MS after 48 h of doxycycline treatment followed by 12 h of MG132 treatment, during which Sox2 expression was predominantly localized in the cytosol (Supplementary Figure S3C).

**Figure 4:**
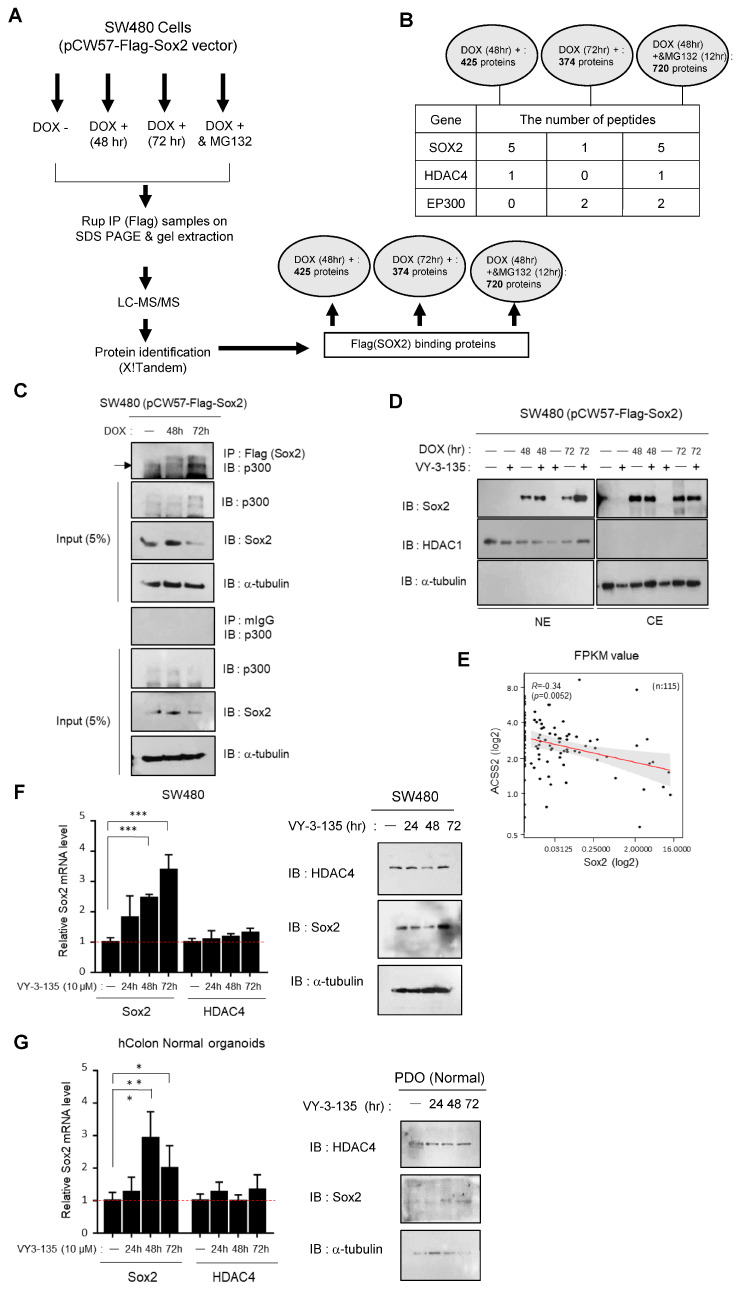
Analysis of Sox2 binding partners and the role of ACSS2-mediated p300 activity during Sox2 degradation. (**A**,**B**) The experimental design for identifying Sox2 binding partners using Flag-immunoprecipitation coupled with LC-MS/MS. Immunoprecipitation (IP) was performed using anti-Flag antibody-conjugated beads or resin to specifically isolate the Flag-tagged Sox2 protein complexes from cell lysates. LC-MS/MS analysis was then carried out to identify the interacting proteins. The number of peptides binding to Flag was quantified using the MS flag tool (https://www.flag-ms.com/, accessed on 30 January 2024) and presented in (**B**). (**C**) A doxycycline-inducible Sox2-overexpressing stable cell line (pCW57-Flag-Sox2) was treated with doxycycline for 48 h and 72 h, followed by pull-down assay using the Flag tag. Then, immunoblotting was performed using a p300 antibody. Mouse IgG was used as a control for the pull-down assay. Full-length blots/gels are presented in Supplementary Figure S7. (**D**) Subcellular localization of Sox2 was determined in nuclear (NE) and cytosolic (CE) fractions of pCW57-Flag-Sox2 cells treated with doxycycline (1 μg/mL) for 48 h or 72 h or co-treated with doxycycline and the ACSS2 inhibitor VY3-135 (10 μM) for 48 h or 72 h. HDAC1 and α-tubulin were used as loading and fractionation controls. Full-length blots/gels are presented in Supplementary Figure S7. (**E**) Correlation analysis of ACSS2 expression and Sox2 expression was performed using RNA-seq data of stage Ⅲ CRC tissues (n = 115, R = 0.34, *p* < 0.0052). (**F**) SW480 cells were treated with VY3-135 (10 μM) for 24, 48, or 72 h. The mRNA and protein expression levels of Sox2 and HDAC4 were analyzed by qPCR and Western blot, respectively. Presented data represent at least two independent experiments. Statistical significance was determined using unpaired Student's *t*-tests (*** *p* < 0.001). Full-length blots/gels are presented in Supplementary Figure S7. (**G**) Human normal colon organoids were treated with VY3-135 (10 μM) for 24, 48, or 72 h. The mRNA levels of Sox2 and HDAC4 were evaluated relative to RPL3A expression (reference gene) using qPCR, and the protein levels of Sox2 and HDAC4 were assessed by immunoblotting using organoid cell lysates. Statistical significance was determined using unpaired Student’s *t*-tests (* *p* < 0.05, ** *p* < 0.005). Full-length blots/gels are presented in Supplementary Figure S7.

The simplified process for identifying Sox2 binding partners using LC-MS/MS is depicted in Figure 4A. The LC-MS/MS analysis identified a total of 428 Sox2 binding partners after 48 h of doxycycline treatment, which decreased to 374 after 72 h of doxycycline treatment. When the cells were treated with doxycycline for 48 h followed by MG132 for 12 h, a total of 720 binding partners were identified. Notably, the results revealed that the binding between Sox2 and HDAC4 increased when Sox2 was overexpressed. Conversely, during Sox2 degradation, the binding between Sox2 and HDAC4 decreased, whereas the binding between Sox2 and p300 increased. Importantly, treatment with MG132 restored the binding between Sox2 and HDAC4, while the binding between Sox2 and p300 was maintained (Figure 4B). To verify that the binding between Sox2 and p300 aligns with the results shown in Figure 4B, we performed immunoprecipitation using Flag-Sox2-expressing SW480 cells. The results showed that despite a decrease in Sox2 expression after 72 h of doxycycline treatment, Sox2 still exhibited strong binding with p300, consistent with the findings of the LC-MS/MS analysis (Figure 4C).

The intracellular metabolic intermediate acetyl-CoA plays a crucial role in p300 activation by providing acetyl groups both for p300 substrates and also for p300 autoacetylation, a critical event that marks p300 activation [24,25]. Acetyl-CoA synthetase 2 (ACSS2) has been identified as an upstream factor that promotes increased activity of CBP, p300/CBP-associated factor, and other histone acetyltransferases, resulting in enhanced histone acetylation [26]. To investigate the potential impact of ACSS2 and p300 acetyltransferase activity on Sox2 expression, we treated cells with the ACSS2 inhibitor VY3-135. As expected, doxycycline and VY3-135 cotreatment for 72 h led to increased Sox2 expression in the nucleus compared with doxycycline treatment alone (Figure 4D). In light of these findings, we examined the differences in ACSS2 expression between SW480 cells and SW620 cells. We found that ACSS2 expression was significantly higher in SW480 cells than in SW620 cells (Supplementary Figure S4A). Moreover, analysis of publicly available Gene Expression Omnibus data (GSE39582) revealed that Sox2 expression is higher in CRC tissues than in normal colon tissues, whereas ACSS2 expression is higher in normal colon tissues than in CRC tissues (Supplementary Figure S4B), suggesting an inverse relationship between Sox2 expression and ACSS2 expression. We next performed RNA sequencing of 115 CRC tumor tissues and analyzed the data in terms of fragments per kilobase of transcript per million mapped fragments (FPKM) values. The analysis revealed a negative correlation between ACSS2 and Sox2 (R = 0.34, *p* < 0.0052), further supporting the inverse relationship between ACSS2 expression and Sox2 expression in CRC (Figure 4E). Taken together, these results suggest that the expression of ACSS2 plays a crucial role in regulating the stability of Sox2 by modulating the histone acetyltransferase activity of p300. These findings highlight the significant roles of HDAC4 and CBP/p300 as binding proteins involved in the regulation of Sox2 expression.

We further investigated the potential contribution of ACSS2 to Sox2 regulation by treating SW480 cells and patient-derived normal colon organoids with the ACSS2 inhibitor VY3-135 and assessed the expression levels of Sox2 and HDAC4 (Figure 4F,G). Following 72 h of VY3-135 treatment, we observed a significant increase in Sox2 expression, while HDAC4 expression remained unchanged. These findings suggest that suppression of p300 acetyltransferase activity due to ACSS2 inhibition rendered HDAC4 unnecessary as a deacetylase for Sox2. Sox2 expression was elevated in the presence of ACSS2 inhibition even in normal colon organoids, which implies that ACSS2 likely plays a role in the regulation of Sox2 expression.

### 3.4. Depletion of HDAC4 Induces Cytoplasmic Shift and Downregulation of Sox2 Expression

**Figure 5:**
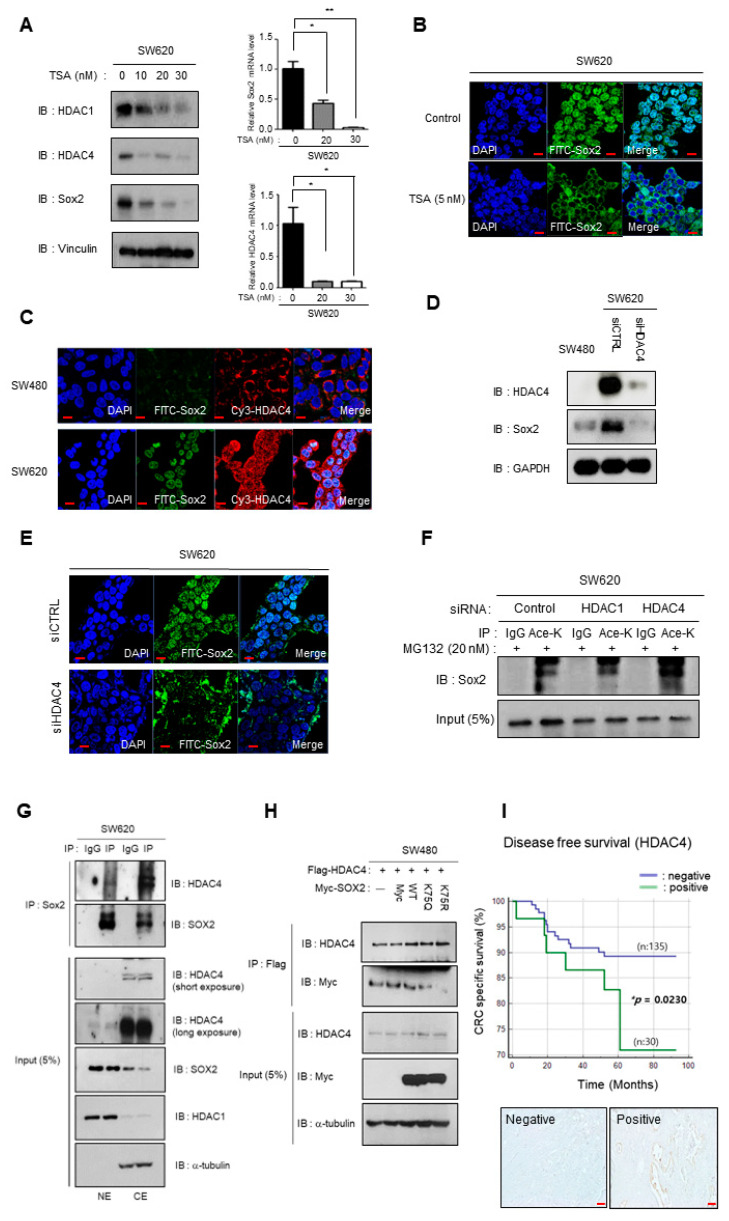
HDAC4 depletion promotes cytoplasmic shift and degradation of Sox2 via increased lysine acetylation. (**A**) SW620 cells were treated with different concentrations of the deacetylase inhibitor trichostatin A (TSA; 10, 20, or 30 nM) for 48 h. The expression of HDAC4, HDAC1, Sox2, and Vinculin was examined by Western blot and qPCR. Data represent three independent experiments. Bar graphs show means ± SEM. Statistical significance was determined using unpaired Student’s *t*-tests (* *p* < 0.05, ** *p* < 0.005). Full-length blots/gels are presented in Supplementary Figure S7. (**B**) SW620 cells were treated with 5 nM TSA for 24 h, followed by immunofluorescence staining with anti-Sox2 (green). Scale bar, 10 μm. (**C**) Immunofluorescence staining was performed using anti-Sox2 (green) and HDAC4 (red) antibodies to visualize their localization in SW480 and SW620 cells. Scale bar, 10 μm. (**D**) SW480 and SW620 cells were transfected with control or HDAC4 siRNA (20 nM) for 48 h. Cell lysates were analyzed by Western blot using anti-HDAC4, Sox2, and GAPDH antibodies. Full-length blots/gels are presented in Supplementary Figure S7. (**E**) SW620 cells were transfected with HDAC4 siRNA (20 nM) for 48 h, and the expression of Sox2 (green) was analyzed by immunofluorescence. Scale bar, 10 μm. (**F**) SW620 cells were transfected with control, HDAC1 or HDAC4 siRNAs (20 nM) for 48 h, with additional MG132 (25 nM) treatment for 24 h. Then, lysates were immunoprecipitated with lysine acetylation antibody, followed by Western blot analysis using anti-Sox2 antibody. Full-length blots/gels are presented in Supplementary Figure S7. (**G**) After fractionation into nuclear (NE) and cytosol (CE) fractions, SW620 cells were subjected to immunoprecipitation using Sox2 antibody. Western blot was performed with anti-HDAC4 and Sox2 antibodies. HDAC1 and GAPDH were used as loading controls for nuclear and cytosolic fractions, respectively. Full-length blots/gels are presented in Supplementary Figure S7. (**H**) SW480 cells were co-transfected with plasmids encoding Flag-HDAC4 and Myc-Sox2 wild-type, K75Q mutant, or K75R mutant to investigate the interaction between Sox2 and HDAC4. After 48 h, cell lysates were immunoprecipitated with anti-Myc antibody, followed by immunoblot analysis using the indicated antibodies. Full-length blots/gels are presented in Supplementary Figure S7. (**I**) Kaplan–Meier curves for disease-free survival analysis were generated based on HDAC4-negative (n:135) and -positive (n:30) expression detected by immunohistochemical staining with anti-HDAC4 antibody in stage Ⅲ primary CRC tissues. Statistical significance between groups was evaluated using log rank test with * *p* < 0.05 as the threshold for significance.

To determine the precise role of HDACs in Sox2 regulation, we treated SW620 cells with the HDAC inhibitor trichostatin A (TSA). Remarkably, TSA treatment led to a dose-dependent reduction in the expression of HDAC1, HDAC4, and Sox2 (Figure 5A). Immunofluorescence analysis showed a cytoplasmic shift and decreased expression of Sox2 upon TSA treatment in SW620 cells (Figure 5B). To delve deeper into the role of histone acetylation, we focused on HDAC1 and HDAC2. Previous studies have shown that HDAC1 is a major HDAC that interacts with Sox2 [27], and a relationship between Sox2 and HDAC4 in CRC has been suggested based on Chip-seq data [28]. Notably, HDAC4 exhibited higher expression levels in SW620 cells than in other CRC cell lines, as evidenced by immunoblotting and qPCR (Supplementary Figure S5A). Consistent with these findings, immunofluorescence and mRNA array analyses demonstrated upregulated expression of HDAC4 in SW620 cells compared with that in SW480 cells (Figure 5C).

To determine whether Sox2 degradation is facilitated by ubiquitination following increased acetylation resulting from a functional decrease in HDAC4 activity, we employed siRNA to deplete HDAC4 in SW620 cells. The depletion of HDAC4 resulted in reduced Sox2 expression accompanied by Sox2 cytoplasmic translocation (Figure 5D,E).

Given K75 acetylation of Sox2 is related to nuclear export [9], we examined the effect of HDAC4 depletion on the acetylation status of K75 in Sox2. Interestingly, HDAC4 depletion led to an increase in K75 acetylation in Sox2, whereas HDAC1 depletion showed no such effect (Figure 5F). Although HDAC1 is a known Sox2 binding partner [27], its expression did not differ significantly between SW480 cells and SW620 cells. Consistent with that, HDAC1 depletion did not alter Sox2 expression in SW620 cells (Supplementary Figure S5C,D), suggesting that HDAC1 is not directly involved in the regulation of Sox2 expression.

To assess the direct interaction between HDAC4 and Sox2 in the nucleus and cytoplasm, we performed immunoprecipitation and Western blot using nuclear and cytosolic fractions. Intriguingly, our results demonstrated that Sox2 interacts with HDAC4 both inside and outside the nucleus (Figure 5G). We further investigated the potential impact of HDAC4 on the acetylation level of K75 in Sox2 using immunoprecipitation assays with acetylation mutants of Sox2. Our findings revealed decreased binding affinity between HDAC4 and the Sox2 K75R mutant, indicating that the K75 residue is a specific site for the interaction between Sox2 and HDAC4 (Figure 5H). These results support our hypothesis that HDAC4 suppresses acetylation at the K75 residue of Sox2, thereby promoting Sox2 expression. With these results, our study presents compelling evidence that by exerting its deacetylase activity, HDAC4 plays a crucial role in regulating the localization and function of Sox2. These findings highlight the importance of HDAC4-mediated deacetylation in maintaining the proper localization and functional integrity of Sox2.

An analysis of public data showed an association between high Sox2 mRNA expression and poor prognosis in CRC (Supplementary Figure S2A). Several studies have demonstrated that high Sox2 mRNA expression is an independent prognostic factor for relapse-free survival in patients with CRC [22,29,30]. In order to evaluate the clinical implications of HDAC4, we used immunohistochemistry to assess HDAC4 expression in 165 primary tumor tissues from patients with CRC (stage Ⅲ) and analyzed the relationship between HDAC4 expression and disease-free survival. The results revealed that patients with HDAC4-positive primary tumors had poorer survival compared with patients with HDAC4-negative primary tumors (Figure 5I), matching the results of the Sox2 expression analysis.

### 3.5. miR29a-Mediated Suppression of HDAC4 Reduces Sox2 Expression

**Figure 6:**
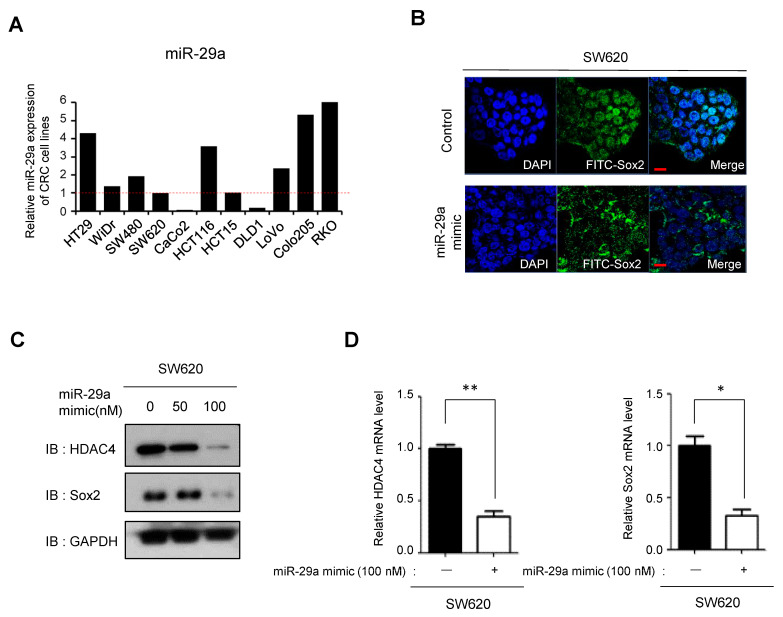
Decreased expression of Sox2 due to miR29a-induced HDAC4 suppression. (**A**) The expression of miR29a in colorectal cancer cell lines was measured by qPCR. Data represent the result of two independent experiments. (**B**) Immunofluorescence study was conducted to observe the localization and expression of Sox2 (green) after transfection of SW620 cells with an miR29a mimic for 72 h. Scale bar, 10 μm. (**C**,**D**) SW620 cells were transfected with scrambled control siRNA or an miR29a mimic for 72 h. The mRNA and protein expression of HDAC4 and Sox2 were quantified by qPCR and Western blot, respectively. Data represent the mean ± SEM of three independent experiments. Statistical significance was determined using unpaired Student’s *t*-tests (* *p* < 0.05, ** *p* < 0.005). Full-length blots/gels are presented in Supplementary Figure S7.

To elucidate miRNA factors associated with the distinct HDAC4 and Sox2 characteristics of SW480 and SW620 cells, we performed mRNA and miRNA array analyses, which revealed differential expression of several mRNAs and miRNAs (Supplementary Figure S6A,B). Among these, miR29a was previously reported to be involved in the regulation of HDAC4 expression, with increased miR29a expression leading to translational repression of HDAC4 through interaction with the HDAC4 3′-untranslated region [31]. We compared the expression levels of miR29a in the SW480 and SW620 cell lines and found that miR29a expression was relatively higher in SW480 cells compared with that in SW620 cells (Figure 6A). Based on these observations, we hypothesized that miR29a could reduce Sox2 expression by suppressing HDAC2. To investigate this hypothesis, we transfected SW620 cells with a miR29a mimic, which resulted in a cytosolic shift of Sox2 and a decrease in Sox2 expression (Figure 6B–D), consistent with the results obtained by HDAC4 depletion (Figure 5D). Collectively, our results suggest that miR29a acts as an upstream regulator of HDAC4 expression and thus exerts control over Sox2 expression.

### 3.6. O-GlcNAcylation and the Related Enzymes OGT and OGA Play Important Roles in Sox2 Regulation

**Figure 7:**
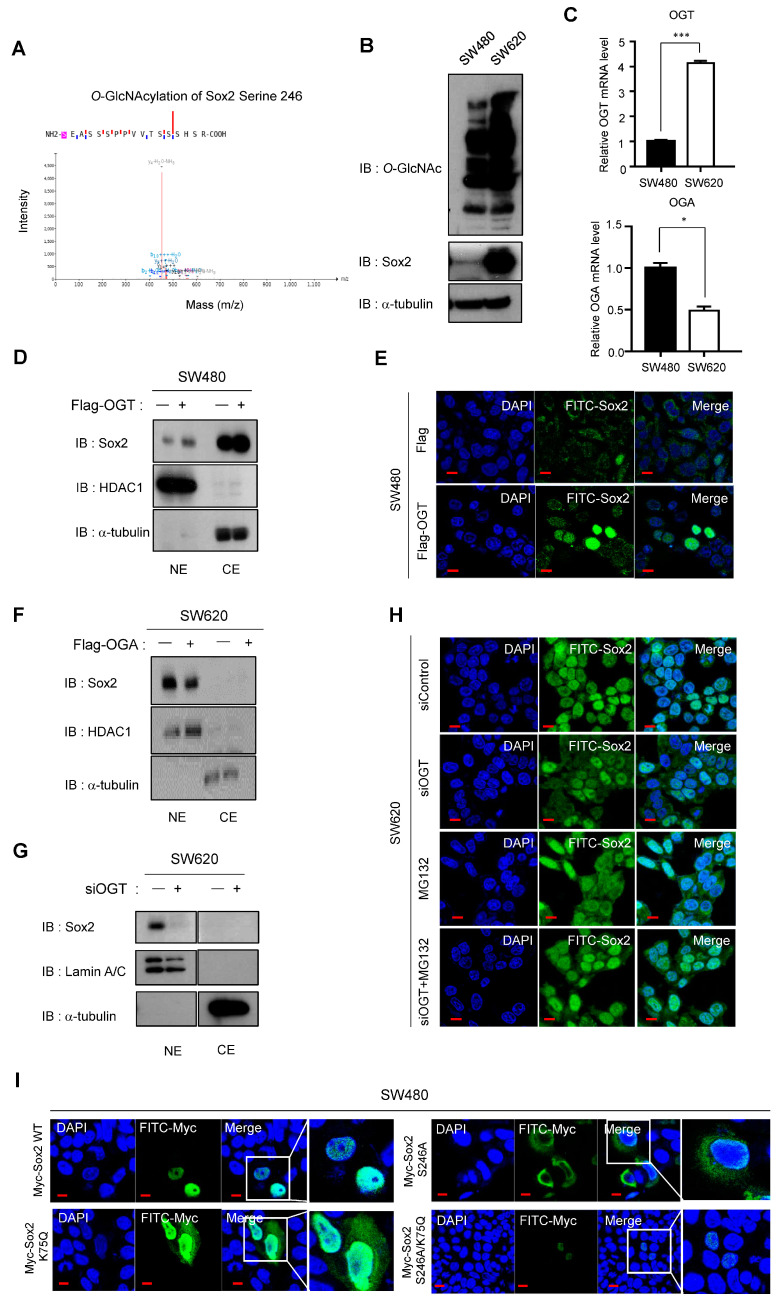
Effects of O-GlcNAcylation by OGT and OGA on Sox2 expression and the combined effects of O-GlcNAcylation and acetylation. (**A**) The O-GlcNAcylation of Sox2 S246 residue in SW480 and SW620 cells was verified by manual inspection of LC-MS/MS spectra. The difference in O-GlcNAcylation of S246 within the O-glycopeptide SEASSSPPVVTSSSHSR sequence with EThcD spectra was confirmed in SW620 cells. Details of the mass spectrometric analysis and database search can be found in the Materials and Methods section. (**B**) Protein lysates of SW480 and SW620 cells were measured by Western blot using anti-O-GlcNAc-specific antibody and Sox2 antibody with α-tubulin as a loading control. Full-length blots/gels are presented in Supplementary Figure S7. (**C**) qPCR measurement of OGT and OGA mRNA levels in SW480 and SW620 cells. Data represent the mean ± SEM of three independent experiments. Statistical significance was determined using unpaired Student’s *t*-tests (* *p* < 0.05, *** *p* < 0.001). (**D**) After transfection with Flag-OGT for 48 h, Western blot was performed using anti-Sox2 antibody. HDAC1 and α-tubulin were used as loading controls for nuclear and cytoplasmic fractions, respectively. Full-length blots/gels are presented in Supplementary Figure S7. (**E**) Immunofluorescence study using anti-Sox2 (green) antibody after transfection with Flag-OGT for 48 h in SW480 cells. Scale bar, 10 μm. (**F**) After transfection with Flag-OGA for 48 h, Western blot was performed using anti-Sox2 antibody. HDAC1 and α-tubulin were used as loading controls for nuclear and cytoplasmic fractions, respectively. Full-length blots/gels are presented in Supplementary Figure S7. (**G**) After transfection with OGT siRNA for 48 h and fractionation of nuclear and cytoplasmic fractions, Western blot analysis was conducted using anti-Sox2 antibody in SW620 cells. Lamin A/C and α-tubulin were used as loading and fractionation controls for nuclear and cytoplasmic fractions, respectively. Full-length blots/gels are presented in Supplementary Figure S7. (**H**) Immunofluorescence study using anti-Sox2 antibody (green) after 48 h of treatment with OGT siRNA (20 nM) in SW620 cells, followed by 24 h of treatment with MG132 (25 nM). Scale bar, 10 μm. (**I**) SW480 cells were transfected with Myc-Sox2 wild-type or K75Q (acetylation mimic), S246A (O-GlcNAc prevention), or S246A/K75Q dual mutant vectors. Immunofluorescence study was performed with anti-Myc (green) antibody. Scale bar, 10 μm.

Several *O*-GlcNAcylation sites have been identified in the Sox2 protein (Supplementary Figure S1). O-GlcNAcylation and expression of *O*-GlcNAc transferase (OGT), the enzyme responsible for catalyzing O-GlcNAcylation, have been reported to be elevated in various cancer types, including CRC [10,32,33,34]. In addition to OGT, *O*-GlcNAcase (OGA) regulates *O*-GlcNAcylation by removing *O*-GlcNAc groups from *O*-GlcNAcylated proteins [35]. We used fusion mass spectrometry to investigate the involvement of *O*-GlcNAcylation in Sox2 regulation and found higher *O*-GlcNAcylation levels, specifically at the S246 residue, in SW620 cells compared with SW480 cells (Figure 7A). Moreover, overall *O*-GlcNAcylation levels were significantly elevated in SW620 cells compared with those in SW480 cells (Figure 7B). Using qPCR to measure mRNA levels, we confirmed that SW620 cells had higher OGT expression and lower OGA expression compared with SW480 cells (Figure 7C).

To elucidate the mechanisms underlying Sox2 regulation by *O*-GlcNAcylation, we overexpressed OGT in SW480 cells and observed increased Sox2 expression in both the nucleus and the cytoplasm (Figure 7D,E). Conversely, overexpression of OGA in SW620 cells resulted in decreased Sox2 expression in the nucleus (Figure 7F). Furthermore, siRNA-mediated depletion of OGT led to reduced Sox2 expression in the nucleus of SW620 cells (Figure 7G,H). Importantly, the siOGT-induced suppression of Sox2 expression was rescued by treatment with MG132, indicating the involvement of *O*-GlcNAcylation in Sox2 stability (Figure 7H).

**Figure 8:**
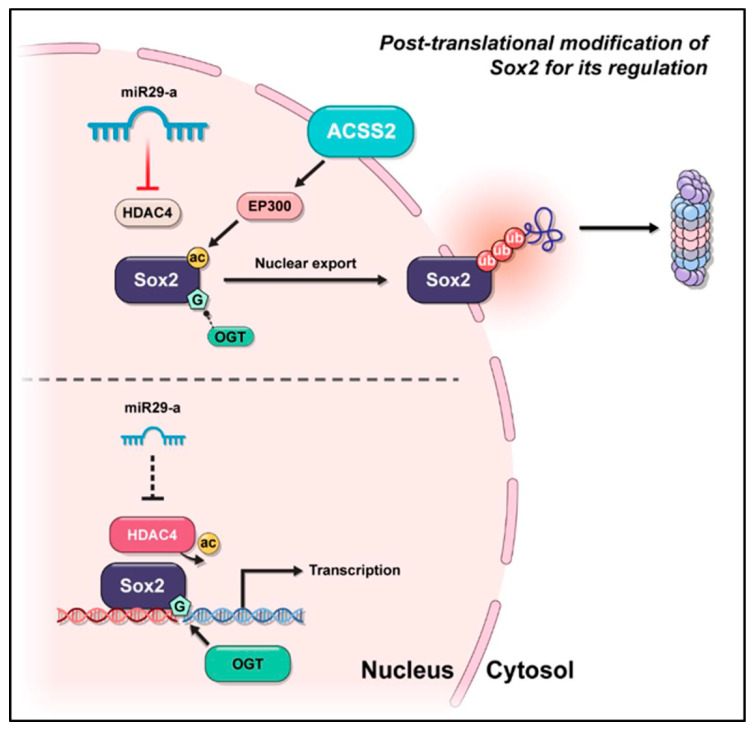
Post-translation modification of Sox2 for its regulation.

Previous studies reported *O*-GlcNAcylation modifications at the S246 residue within the transactivation domain of Sox2, as well as acetylation at K75. To examine the individual and combined roles of these PTMs in CRC, we generated a Sox2 S246A mutant (*O*-GlcNAcylation-null) and a Sox2 K75Q/S246A double mutant (acetylation mimic/*O*-GlcNAcylation-null). Transfection of the Sox2 K75Q acetylation-mimic mutant into SW480 cells resulted in increased cytoplasmic localization of Sox2 compared with that in cells expressing wild-type Sox2. Conversely, SW480 cells transfected with the Sox2 S246A mutant showed reduced Sox2 expression in the nucleus, while SW480 cells transfected with the K75Q/S246A double mutant exhibited minimal Sox2 expression in both the nucleus and the cytoplasm (Figure 7I). These findings suggest an additive suppressive effect of the acetylation-mimic mutation and the *O*-GlcNAcylation-null mutation on Sox2 expression. In summary, we investigated PTMs of Sox2, including acetylation regulated by ACSS2-p300 and miR29a-HDAC4, as well as *O*-GlcNAcylation, play a role in its regulation (Figure 8).

## 4. Discussion

Sox2 is a critical pluripotent factor involved in stem cell self-renewal, reprogramming, and homeostasis. Regulation of Sox2 expression occurs through a complex network of transcriptional controls and PTMs. Aberrant Sox2 expression resulting from gene amplification, genetic alterations, or protein overexpression is frequently observed in various cancers and has been associated with poor prognosis in patients with CRC [30]. In general, Sox2 is known to promote proliferation, invasion, metastasis, stemness, and drug resistance in cancer cells, making it an attractive target for anticancer therapies. Therefore, it is necessary to gain a comprehensive understanding of Sox2 regulation, its association with multiple signaling pathways in cancer, and its role in tumorigenesis and drug resistance.

Acetylation of Sox2 has been extensively studied in embryonic stem cells and somatic cells, where it plays a crucial role in stem cell function and somatic cell reprogramming. SIRT1, a NAD-dependent deacetylase, maintains Sox2 at low levels in embryonic stem cells, and its deacetylase activity is linked to somatic cell reprogramming [36]. Furthermore, SIRT1-mediated deacetylation contributes to maintaining Sox2 protein in the nucleus of bone marrow mesenchymal stem cells [37]. Instead, we discovered that p300/CBP acetyltransferase activity acetylates the K75 residue of Sox2, leading to Sox2 nuclear export and subsequent polyubiquitination and proteasomal degradation [9]. In addition, we found that upstream regulation of Sox2 acetylation involves the ACSS2/p300 axis. Bulusu, Vinay et al. showed that equilibration between nuclear and cytosolic acetyl-CoA is limited, and ACSS2 nuclear translocation increases p300/CBP activity [26]. In gastric cancer and CRC, unlike other types of cancer, lower expression of ACSS2 is associated with tumor progression and metastasis [38,39]. In the normal colon, ACSS2 synthesizes acetyl-CoA from acetate, which is produced by the fermentation of dietary fibers by gut microflora. However, in CRC, expression of ACSS2 is reduced because of the reliance of CRC cells on glycolysis as their primary energy source [40,41]. Our experimental data suggest that depletion or inhibition of ACSS2 inhibits the enzymatic functions of p300/CBP on the acetylation residues of Sox2, causing Sox2 to be retained in the nucleus and implicating the ACSS2/p300 axis in Sox2 regulation.

An intriguing possibility is the potential influence of microsatellite status on the loss of ACSS2 expression in CRC. ACSS2 contains short repeats of 3–7 mononucleotides (C, G, or A) within its coding regions. These repetitive sequences can be prone to frame-shift mutations, leading to a loss-of-function phenotype. This phenomenon is particularly relevant in cancers exhibiting microsatellite instability (MSI), such as MSI-high gastric cancers and CRCs [40,41]; however, no clinical data have yet established a direct relationship among ACSS2, MSI-high tumors, and Sox2 expression. Further investigation is needed to elucidate the precise mechanisms underlying ACSS2 downregulation in CRC and its potential connection to microsatellite status.

The observed increase in Sox2 stability and expression upon modulation of Sox2 acetylation by ACSS2 inhibitor in both normal and cancer in vitro models underscores the significance of our study. However, considering the diverse roles of ACSS2 in vivo, further investigations are warranted to ascertain potential differential effects or alterations induced by ACSS2 inhibitor usage in vivo. Additionally, the necessity to validate histone acetylation changes highlights the importance of future research. These findings contribute to our understanding of the intricate mechanisms underlying Sox2 regulation and its implications in both normal and cancer conditions.

Several HDACs are known to catalyze deacetylation. Among them, HDAC4, a member of the class IIa histone deacetylases, exhibits nucleocytoplasmic shuttling [42], is implicated in various cancers, and is involved in histone deacetylation leading to transcriptional repression. In HCT116 colon cancer cells, downregulation of HDAC4 expression was shown to inhibit growth, induce apoptosis, reduce xenograft tumor growth, and increase p21 transcription [43]. A Chip-Seq analysis performed by Xuefeng Fang et al. revealed a potential link between HDAC4 and Sox2 expression, suggesting that HDAC4 may play a role in connecting histone modification and Sox2 regulation [28]. In line with those findings, our study provides the first direct evidence of HDAC4-mediated Sox2 regulation. Moreover, in terms of clinical outcomes, we found that HDAC4 expression and Sox2 expression were both negatively associated with survival in CRC, which matches a previous report that increased HDAC4 expression was associated with poor prognosis in esophageal cancer [44].

MiRNAs are small noncoding RNAs that play a role in post-transcriptional gene regulation and have been implicated in cancer progression [45,46]. In a previous study, TGF-β1 was reported to reduce miR29a expression, promoting tumorigenicity and metastasis of cholangiocarcinoma by upregulating HDAC4 [31,47]. Our results show that miR29a negatively regulates Sox2 expression by suppressing HDAC4 and HDAC4-induced deacetylation in CRC. This highlights the role of miRNAs in the post-transcriptional regulation of Sox2 and adds to the understanding of Sox2 regulation in cancer progression.

*O*-GlcNAcylation is a process in which GlcNAc from UDP-GlcNAc is transferred to protein serine or threonine residues, reflecting the nutritional status of cells [48]. In pancreatic cancer, OGT is markedly overexpressed and catalyzes *O*-GlcNAcylation at the S246/248 residues of Sox2, stabilizing Sox2 in the nucleus and promoting self-renewal of tumor cells [13]. We observed a similar pattern in CRC, where high OGT levels were associated with *O*-GlcNAcylation and nuclear stability of Sox2. Furthermore, we found that simultaneous activation of acetylation and inhibition of *O*-GlcNAcylation led to near-complete suppression of Sox2 in CRC cells, suggesting that the additive effects of these PTMs on Sox2 suppression might be used in cancer treatment.

Other types of PTMs have also been reported to regulate Sox2 activity in different organs and tumors. Van Hoof et al. reported that phosphorylation of Sox2 at consecutive serine sites (S249, S250, and S251) leads to SUMOylation and functional effects in human embryonic stem cells [49]. Sox2 can be phosphorylated by AKT1 [50] and Aurora kinase A [51], which impedes its transcriptional activity and triggers its ubiquitination and subsequent degradation; however, our PTM analysis in CRC cells lines did not reveal any evidence of phosphorylation contributing to the expression of Sox2. In another paper, it was reported that a CpG island in the Sox2 promoter that was hypomethylated in high Sox2-expressing malignant gliomas compared with normal tissues. They further showed that in Sox2-negative glioma cell lines, treatment with the DNA methyl-transferase inhibitor, 5-azacitidine (5-AZA), resulted in increased Sox2 expression at the mRNA and protein levels, whereas treatment with TSA had no such effect [52]. In contrast to those findings, we found that treatment with 5-AZA did not induce Sox2 expression in CRC cells with low Sox2 expression, suggesting no involvement of methylation in Sox2 regulation in CRC (Supplementary Figure S5E).

## 5. Conclusions

Various reports suggest that the transcription factor Sox2, a master regulator of embryonic and induced pluripotent stem cells, drives cancer stemness, fuels tumor initiation, and contributes to tumor aggressiveness via major drug resistance mechanisms. Investigations into the mechanistic basis of Sox2 regulation, with a focus on the generation of cancer stem cells and chemoresistance, might lead to new therapeutic approaches involving Sox2-targeted treatment. Our findings provide a comprehensive picture of how Sox2 expression is regulated in CRC through acetylation, deacetylation, and *O*-GlcNAcylation modulated by ACSS2/p300, miR29a/HDAC4, and their interactive effects.

## Data Availability

Data are contained within this article.

## Supplementary Materials

The following supporting information can be downloaded at: https://www.mdpi.com/article/10.3390/cancers16051035/s1, Figure S1. Post-translational modification sites of Sox2. Figure S2. Expression of Sox2 in normal and colorectal tumors and its association with poor survival in patients with CRC. Figure S3. Time-dependent Sox2 degradation through the proteasomal pathway and inability to sustain Sox2 stability. Figure S4. ACSS2 and Sox2 expression in CRC cells (SW480 and SW620) and human CRC tissues. Figure S5. Lack of significant change in Sox2 expression upon HDAC1 suppression and methylation inhibition. Figure S6. mRNA and miRNA array analyses in SW480 and SW620 cells. Figure S7. Immunoblotting gel images. Table S1. Comprehensive list of antibodies, biological samples, agents, cell lines, and oligonucleotides used.
