# Supplementary material for: A Comprehensive Understanding of Post-Translational Modification of Sox2 via Acetylation and O-GlcNAcylation in Colorectal Cancer
Source: Cancers (Basel). 2024 Mar 3;16(5):1035. doi: 10.3390/cancers16051035 (PMC10931190; doi:10.3390/cancers16051035)

Cancers

Supplemental information

A comprehensive understanding of post-translational modification of Sox2 via acetylation and *O*-GlcNAcylation in colorectal cancer

Yoojeong Seo, Dong Keon Kim, Jihye Park, Soo Jung Park, Jae Jun Park, Jae Hee Cheon, Tae Il Kim

Supplementary figures related to manuscript:

A comprehensive understanding of post-translational modification of Sox2 via acetylation and *O*-GlcNAcylation in colorectal cancer

Yoojeong Seo *et al*., 2024

Overview

Supplementary Figures and Legends

Figure S1. Post-translational modification sites of Sox2.

Figure S2. Expression of Sox2 in normal and colorectal tumors and its association with poor survival in patients with CRC.

Figure S3. Time-dependent Sox2 degradation through the proteasomal pathway and inability to sustain Sox2 stability.

Figure S4. ACSS2 and Sox2 expression in CRC cells (SW480 and SW620) and human CRC tissues.

Figure S5. Lack of significant change in Sox2 expression upon HDAC1 suppression and methylation inhibition.

Figure S6. mRNA and miRNA array analyses in SW480 and SW620 cells.

Figure S7. Immunoblotting gel images

Supplementary Figure S1.

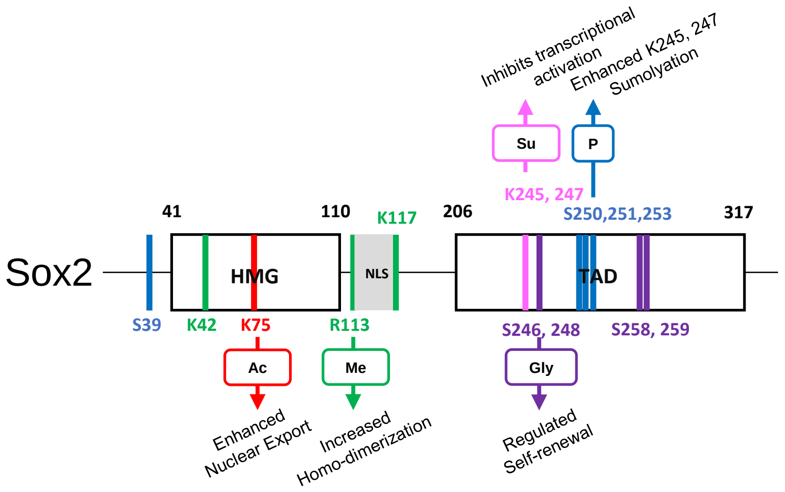

Supplementary Figure S1. Post-translational modification sites of Sox2.

PTMs of Sox2 are shown with their functional effects. Data obtained from studies of human and mouse Sox2 are combined on the mouse amino acid sequence. Ac: Acetylation (Red), Me: methylation (Green), Su: SUMOlyation (Pink), P: Phosphorylation (Blue), and Gly: *O*-GlcNAc glycosylation (Purple).

Supplementary Figure S2.

A

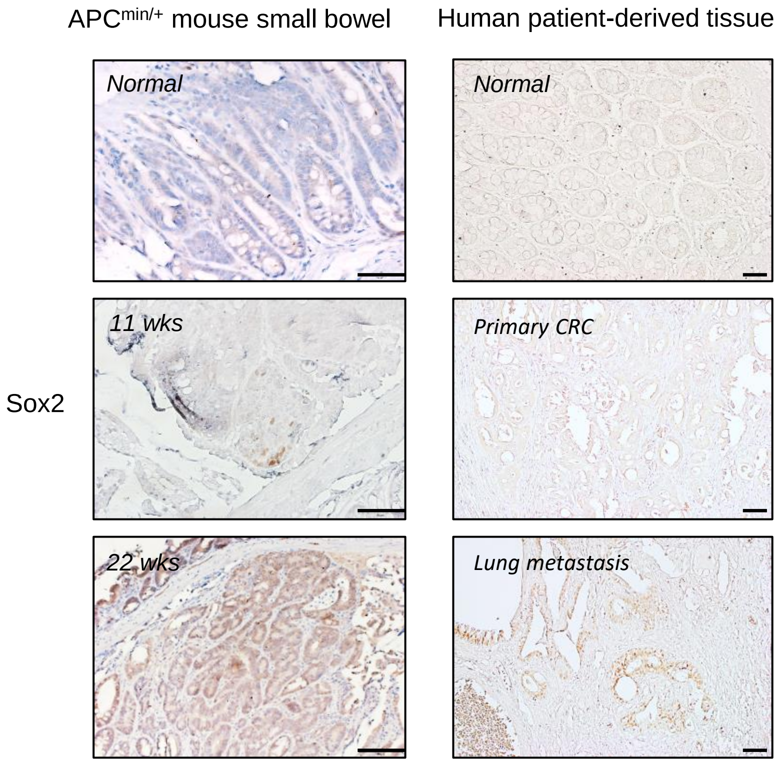

B

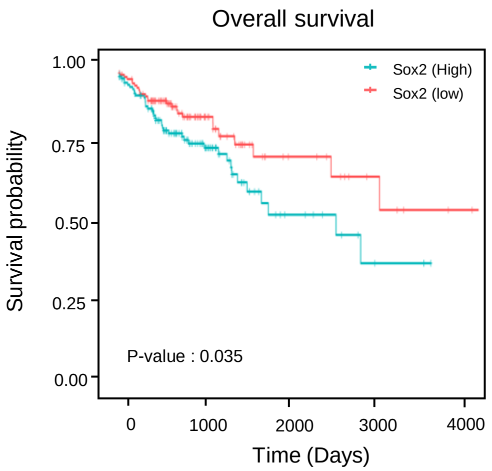

Number at risk

	0	1000	2000	3000	4000
Sox2-low group	162	50	13	6	2
Sox2-high group	162	50	11	3	0

Time (Days)

Supplementary Figure S2. Expression of Sox2 in normal and colorectal tumors and its association with poor survival in patients with CRC.

(A) Sox2 expression was examined in normal tissue and small bowel tumors of APC$^{min/+}$ mice at different ages (11 weeks and 22 weeks) (Left). Scale bar, 400µm. Additionally, Sox2 staining was performed on normal colon tissue, primary CRC tissue, and metastatic tissue of CRC (Right). Scale bar, 200 µm.

(B) An overall survival graph was generated using data from The Cancer Genome Atlas comparing 226 patients with high Sox2 expression and 233 patients with low Sox2 expression in CRC.

Supplementary Figure S3.

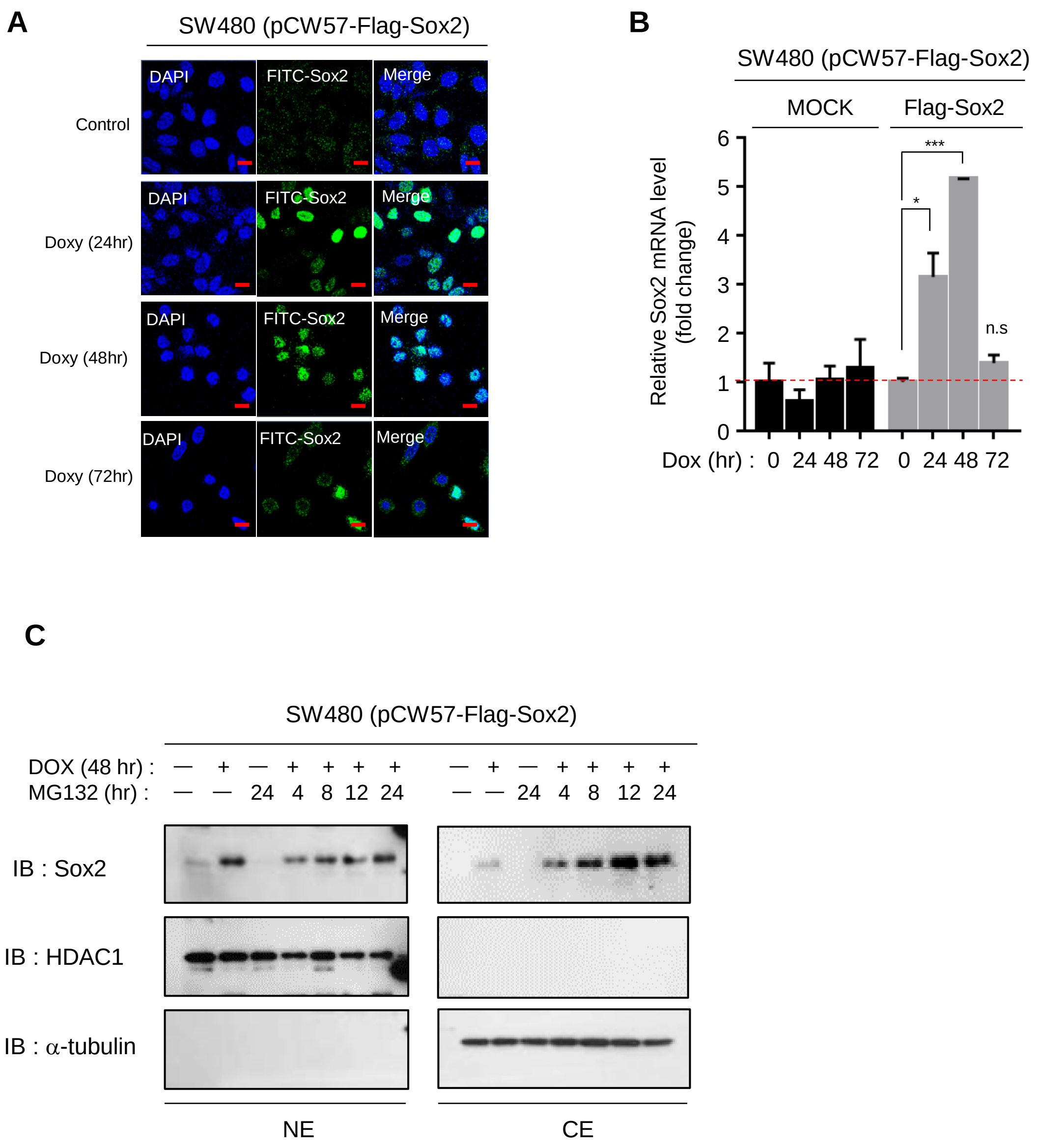

Supplementary Figure S3. Time-dependent Sox2 degradation through the proteasomal pathway and inability to sustain Sox2 stability

(A) A doxycycline-inducible Sox2 stable cell line (pCW57-Flag-Sox2) was treated with doxycycline at a concentration of 1 µg/ml for 24, 48, or 72 h. Immunofluorescence staining was performed using anti-Sox2 antibody (green). Scale bar represents 10 µm.

(B) Following doxycycline treatment as described above, RNA was extracted to measure the mRNA levels of Sox2 using qPCR. The data shown are representative of three independent experiments. The bar graphs represent the means ± SEM. Statistical significance between groups was determined using Student's t-tests (*p<0.05, ***p<0.001).

(C) After treating the doxycycline-inducible Sox2 stable cell line with doxycycline for 48 h, cells were further treated with MG132 (10 nM) for 4, 8, 12, or 24 h. The cells were then fractionated into nuclear (NE) and cytosolic (CE) fractions. Western blot was performed using anti-Sox2 antibody with HDAC1 and α-tubulin used as loading controls for the nuclear and cytosolic fractions, respectively. Full-length blots/gels are presented in Supplementary Figure 7.

Supplementary Figure S4.

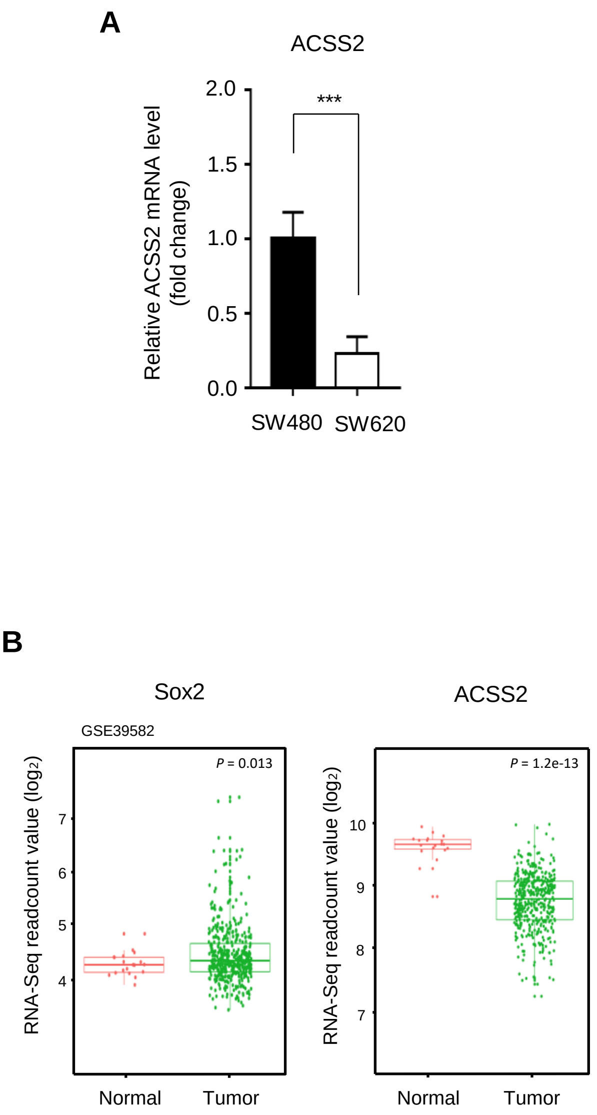

Supplementary Figure S4. ACSS2 and Sox2 expression in CRC cells (SW480 and SW620) and human CRC tissues.

(A) The mRNA expression levels of ACSS2 were analyzed in SW480 and SW620 cells. The bar graphs represent the means ± SEM. Statistical significance between groups was determined using Student's t-tests (***p<0.001).

(B) In the GSE39582 public dataset, the expression of Sox2 and ACSS2 in colon tumor tissues was compared with that in normal colon tissues. The bar graphs represent the means ± SEM. Statistical significance between groups was determined using Student's t-tests (*p<0.05, ***p<0.001).

Supplementary Figure S5.

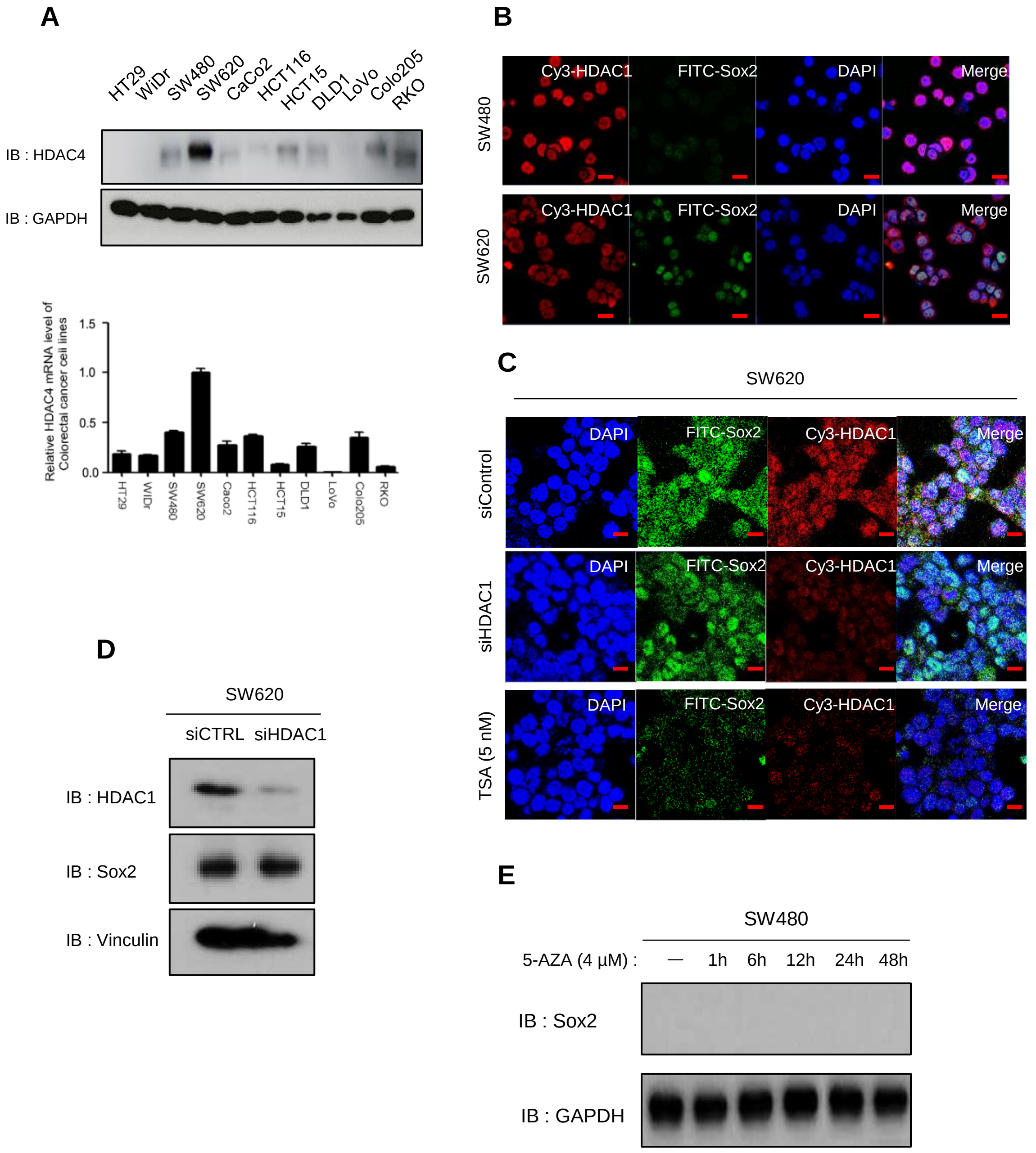

Supplementary Figure S5. Lack of significant change in Sox2 expression upon HDAC1 suppression and methylation inhibition.

(A) HDAC4 expression was measured by western blot using anti-HDAC4 antibody and qPCR in CRC cell lines. Full-length blots/gels are presented in Supplementary Figure 7.

(B) Immunofluorescence study was conducted using antibodies against Sox2 (green) and HDCAC1 (red) in SW480 and SW620 cells. Scale bar: 10 μm.

(C) SW620 cells were treated with 5 nM TSA for 24 h and HDAC1 siRNA for 48 h. Immunofluorescence staining was performed using antibodies against Sox2 (green) and HDAC1 (red). Scale bar: 10 μm.

(D) SW620 cells were transfected with control or HDAC1 siRNA for 48 h. Lysates were subjected to western blot analysis using antibodies against Sox2, HDAC1, and vinculin. Full-length blots/gels are presented in Supplementary Figure 7.

(E) SW480 cells were treated with a DNA methyltransferase inhibitor, 5-AZA for 1, 6, 12, 24, or 48 h. The expression of Sox2 and GAPDH was detected by western blot. Full-length blots/gels are presented in Supplementary Figure 7.

Supplementary Figure S6.

A

SW480	SW620	log2 ratio	Gene Accession	Gene Symbol	Gene Description
5.174805	8.611607	3.436802	NM_003106	Sox2	SRY (sex determining region Y)-box 2
7.208493	8.26747	1.058977	NM_006037	HDAC4	histone deacetylase 4
9.38695	7.525474	-1.861476	NM_018677	ACSS2	acyl-CoA synthetase short-chain family member 2
10.68762	11.14954	0.46192	NM_004964	HDAC1	histone deacetylase 1
11.08322	10.90179	-0.18143	NM_003400	XPO1	exportin 1 (CRM1 homolog, yeast)
10.37297	10.1799	-0.19307	NM_181672	OGT	O-linked N-acetylglucosamine (GlcNAc) transferase (UDP-N-acetylglucosamine:polypeptide-N-acetylglucosaminyl transferase)
3.87165	4.24024	0.36859	NM_058165	MOGAT1	monoacylglycerol O-acyltransferase 1
3.810151	4.135551	0.3254	NM_025098	MOGAT2	monoacylglycerol O-acyltransferase 2
5.482756	5.364568	-0.118188	NM_178176	MOGAT3	monoacylglycerol O-acyltransferase 3

B

Genes	log2 (Fold Change)
Up-regulated (SW620)	
hsa-mir-194-2 // hsa-mir-194-1	4.299
hsa-mir-192	4.158
hsa-mir-375	2.468
hsa-mir-210	1.598
hsa-mir-151	1.531
hsa-mir-17	1.421
hsa-mir-181b-1 // hsa-mir-181b-2	1.391
hsa-mir-200b	1.383
hsa-mir-181a-1 // hsa-mir-181a-2	1.257
hsa-mir-200a	1.208
hsa-mir-18a	1.183
hsa-mir-181a-1 // hsa-mir-181a-2	1.153
hsa-mir-140	1.092
hsa-mir-361	1.082
hsa-mir-19a	1.044
Down-regulated (SW620)	
hsa-mir-886	-3.113
hsa-mir-1308	-2.536
hsa-mir-200c	-2.360
hsa-mir-424	-2.270
hsa-mir-503	-2.213
hsa-mir-146a	-1.950
hsa-mir-10a	-1.687
hsa-mir-638	-1.612
hsa-mir-138-2 // hsa-mir-138-1	-1.566
hsa-mir-29a	-1.501

Supplementary Figure S6. mRNA and miRNA array analyses in SW480 and SW620 cells.

(A) mRNA levels were assessed using the Affymetrix GeneChip® Human Gene 1.0 ST array in SW480 and SW620 cells. The yellow-highlighted regions represent genes that showed upregulation in SW620 cells.

(B) miRNA expression were compared between SW480 and SW620 cells using the Affymetrix GeneChip® Human Gene 1.0 ST miRNA assay.

Supplementary Figure S7.

(Figure 1 B)

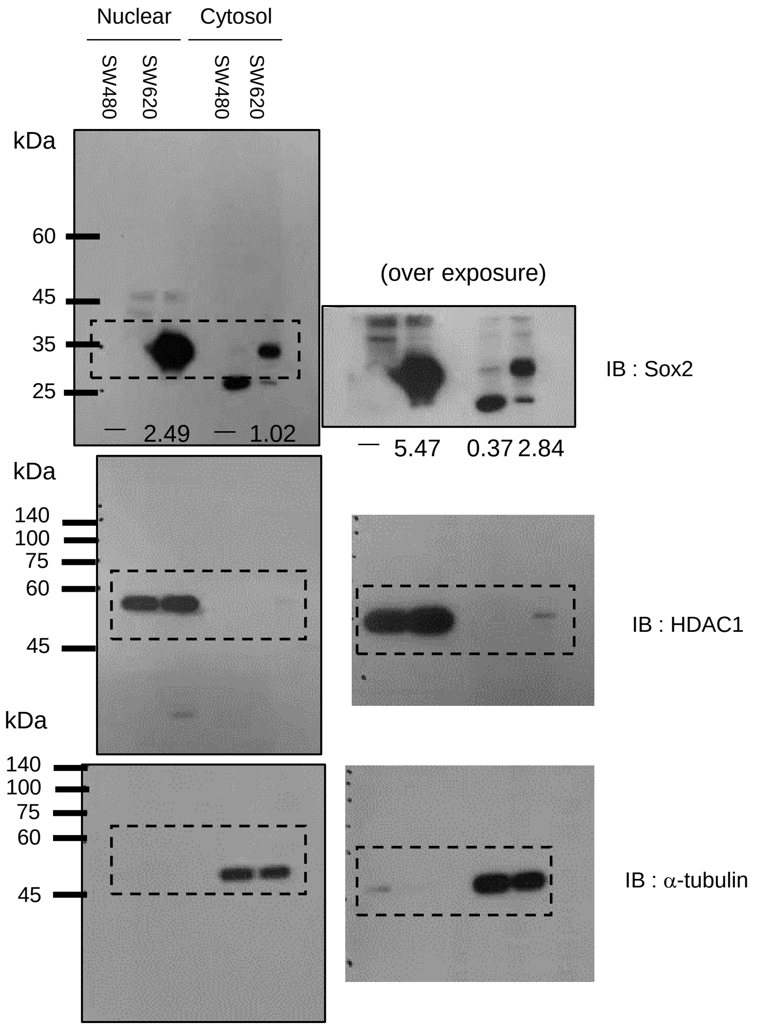

(Figure 1 C)

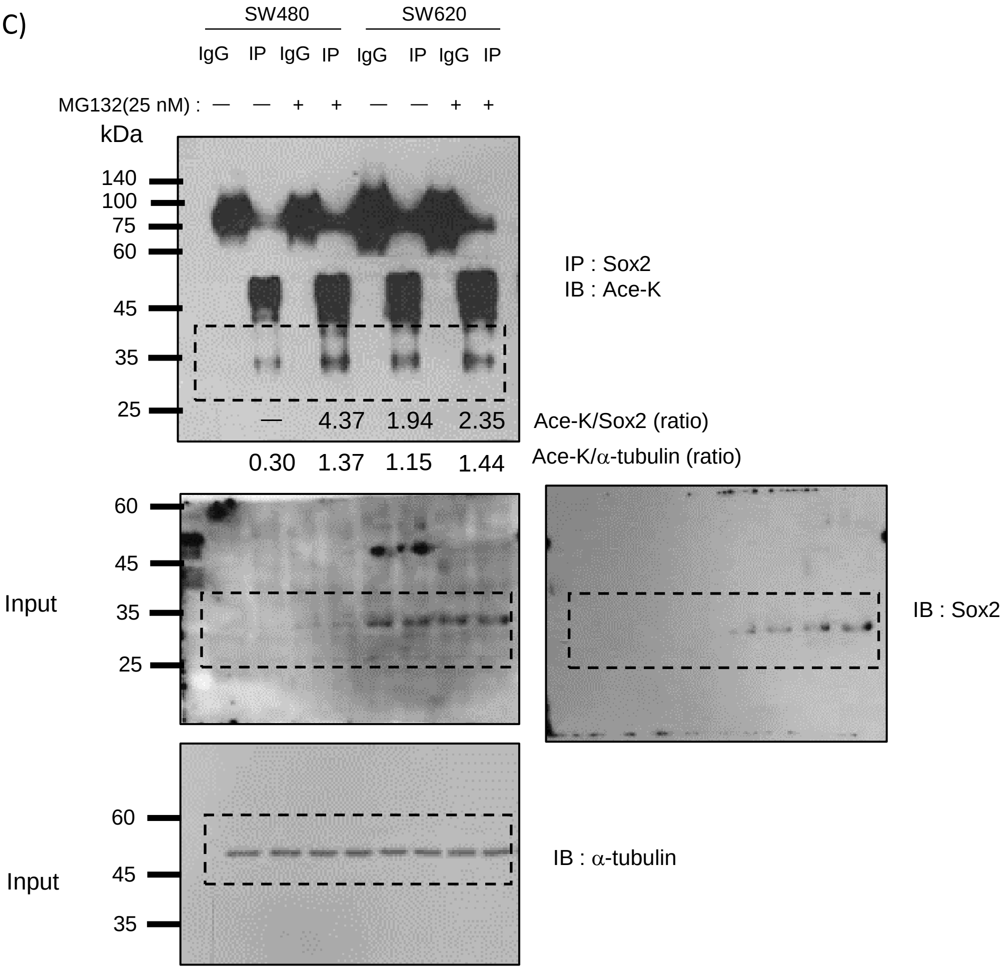

Supplementary Figure S7.

(Figure 2 B)

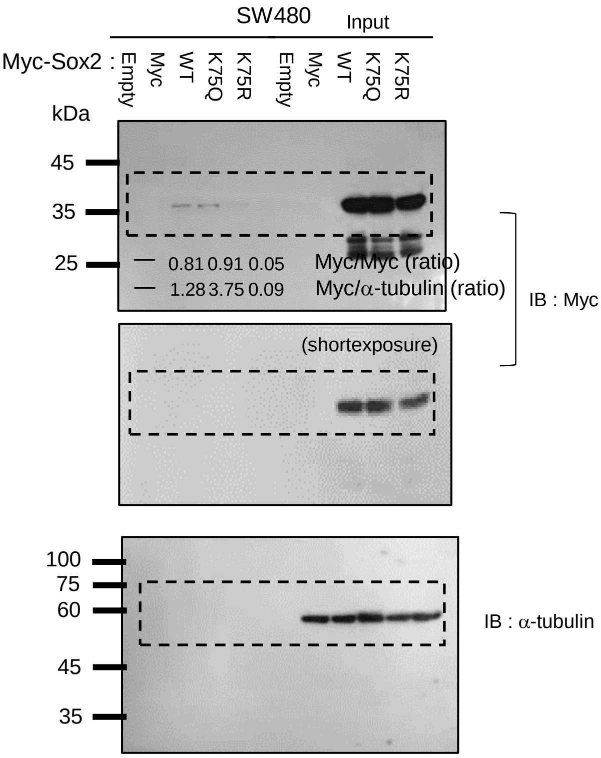

(Figure 2 C)

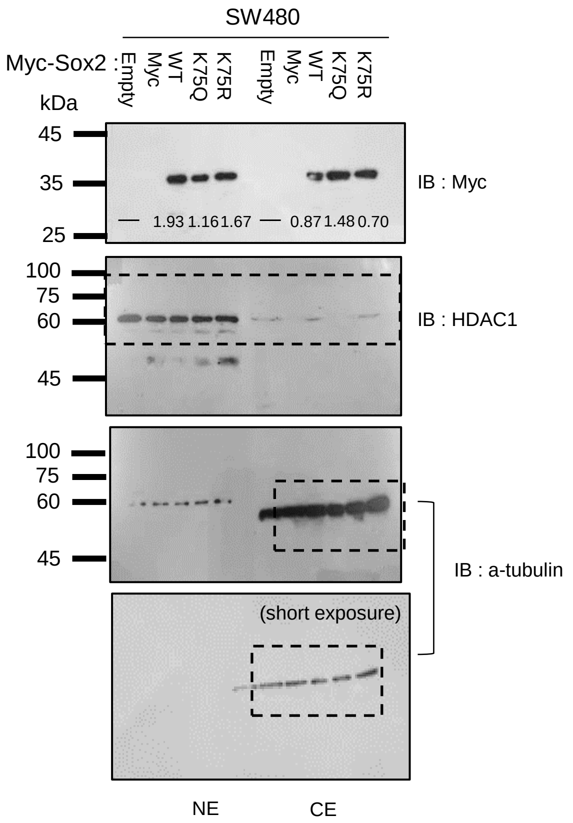

(Figure 3 A)

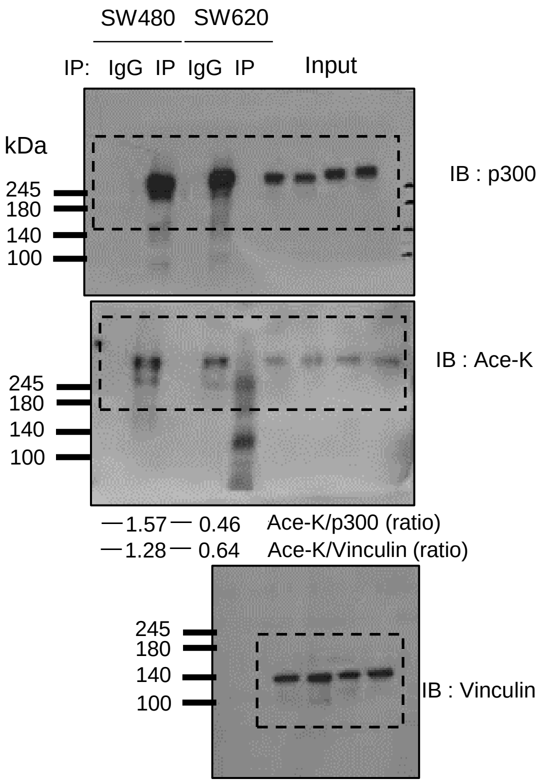

Supplementary Figure S7.

(Figure 3 B)

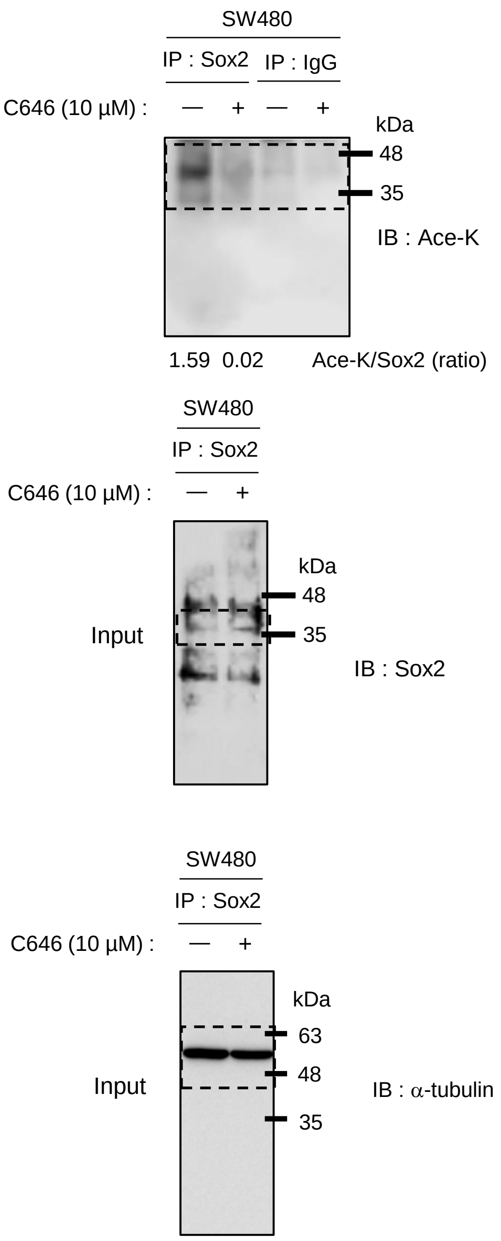

(Figure 3 C)

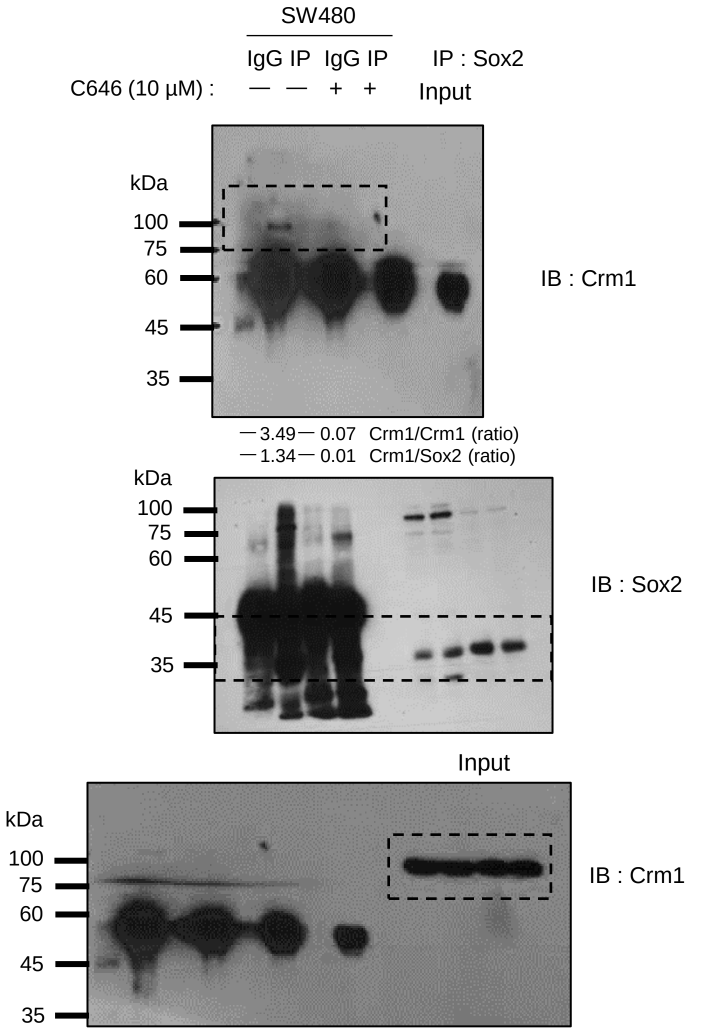

(Figure 3 D)

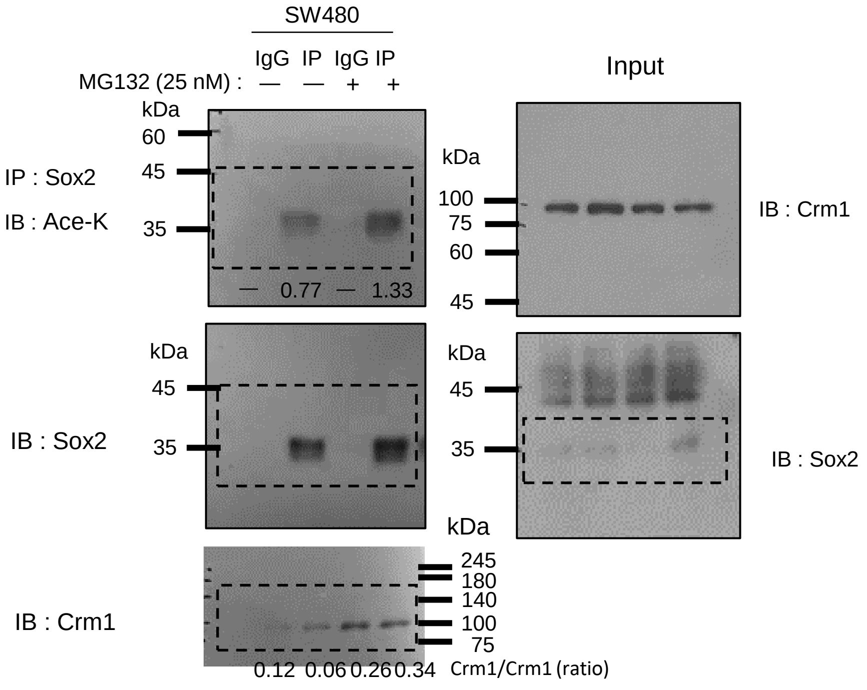

Supplementary Figure S7.

(Figure 4 C)

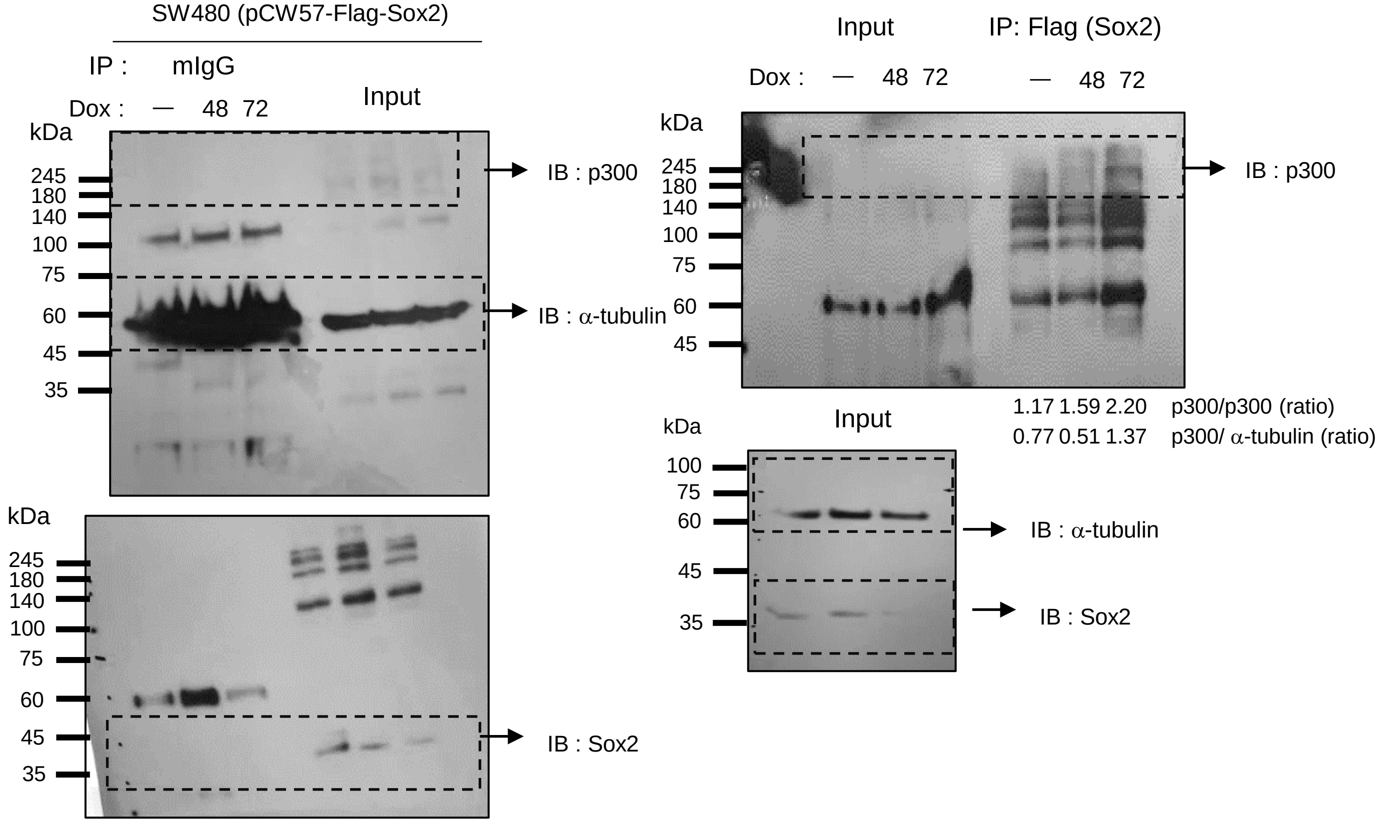

Supplementary Figure S7.

(Figure 4 D)

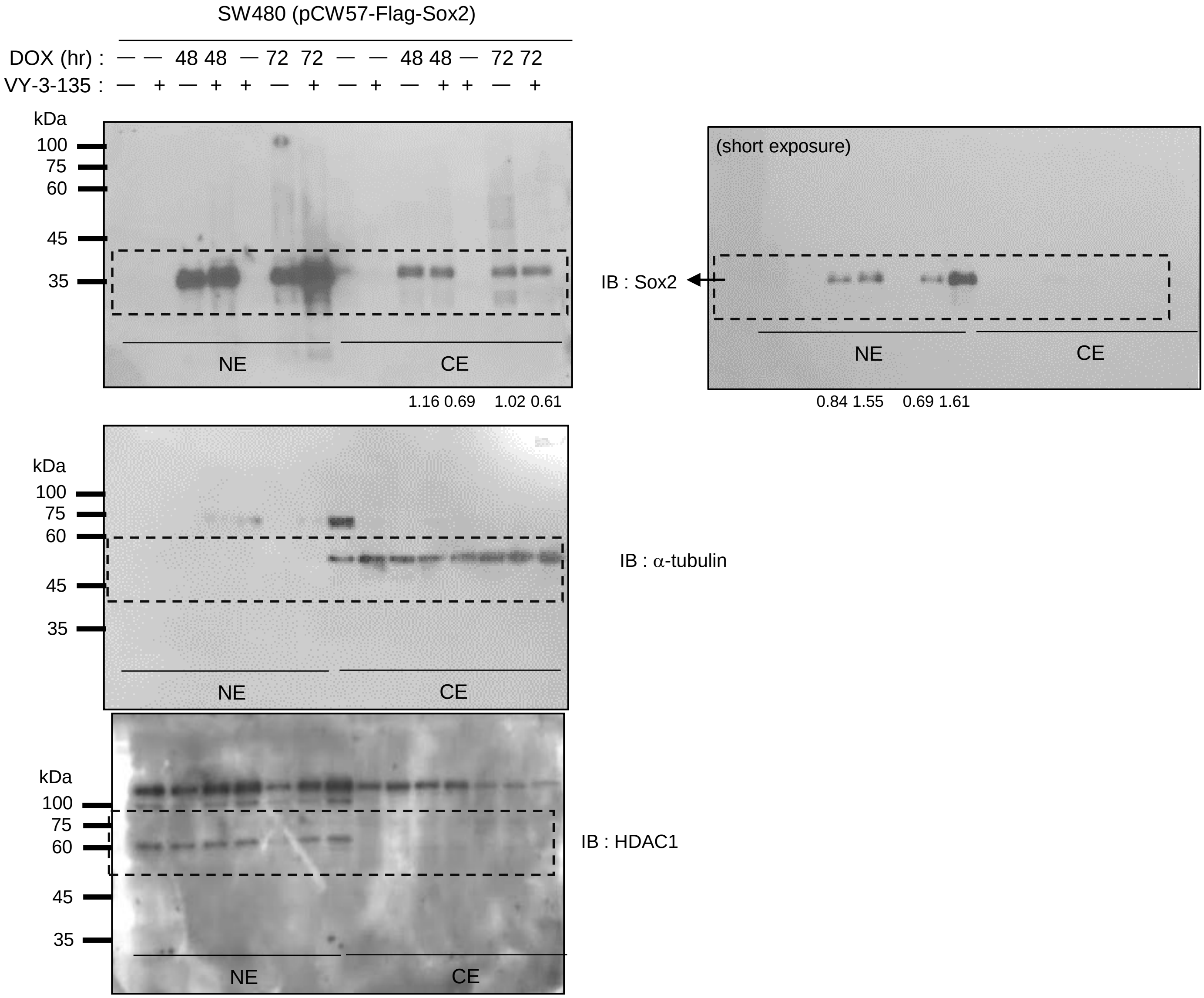

(Figure 4 F)

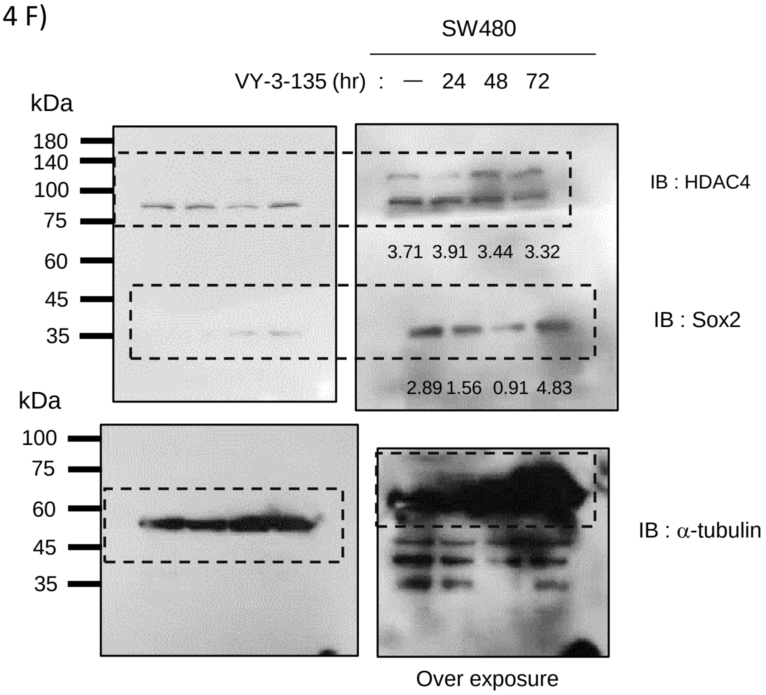

Supplementary Figure S7.

(Figure 4 G)

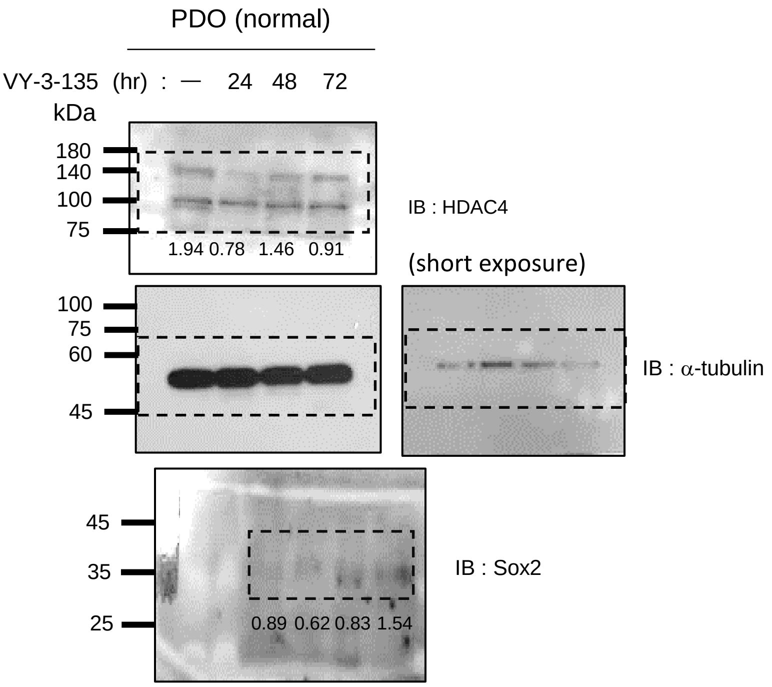

(Figure 5 A)

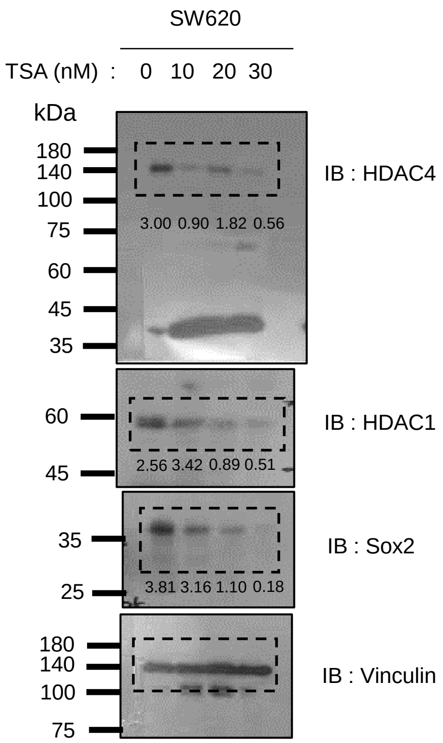

(Figure 5 D)

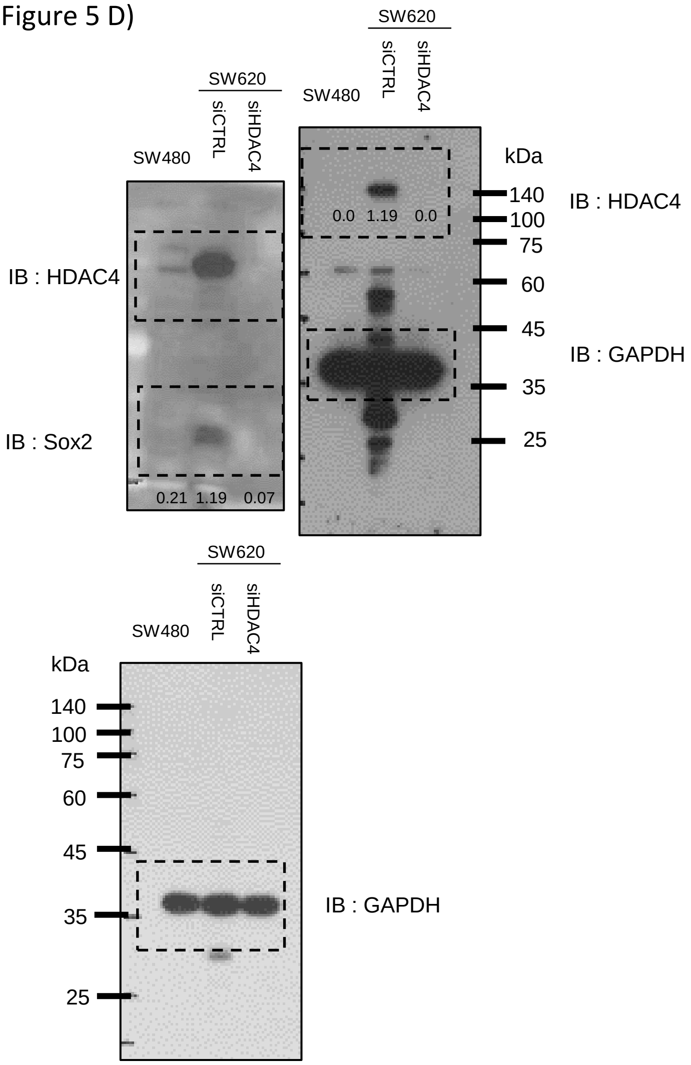

Supplementary Figure S7.

(Figure 5 F)

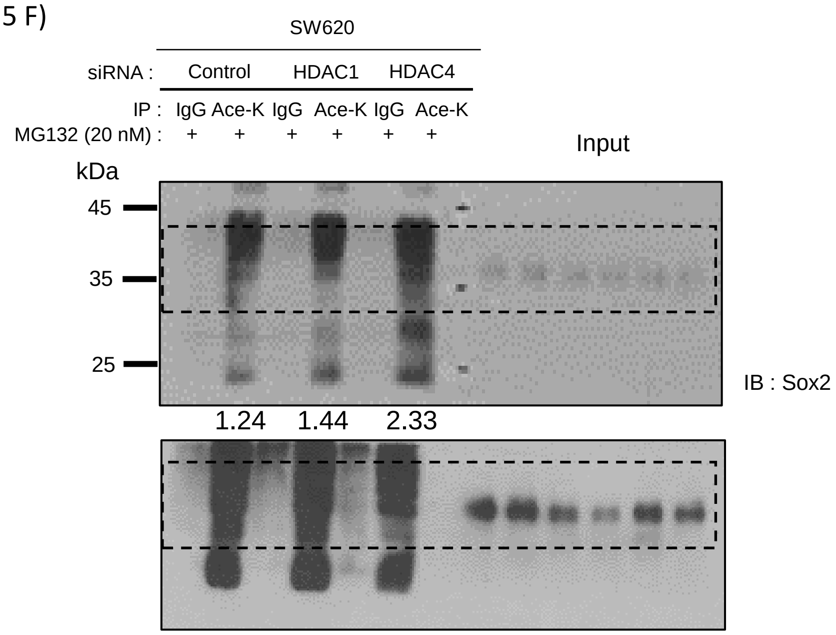

(Figure 5 G)

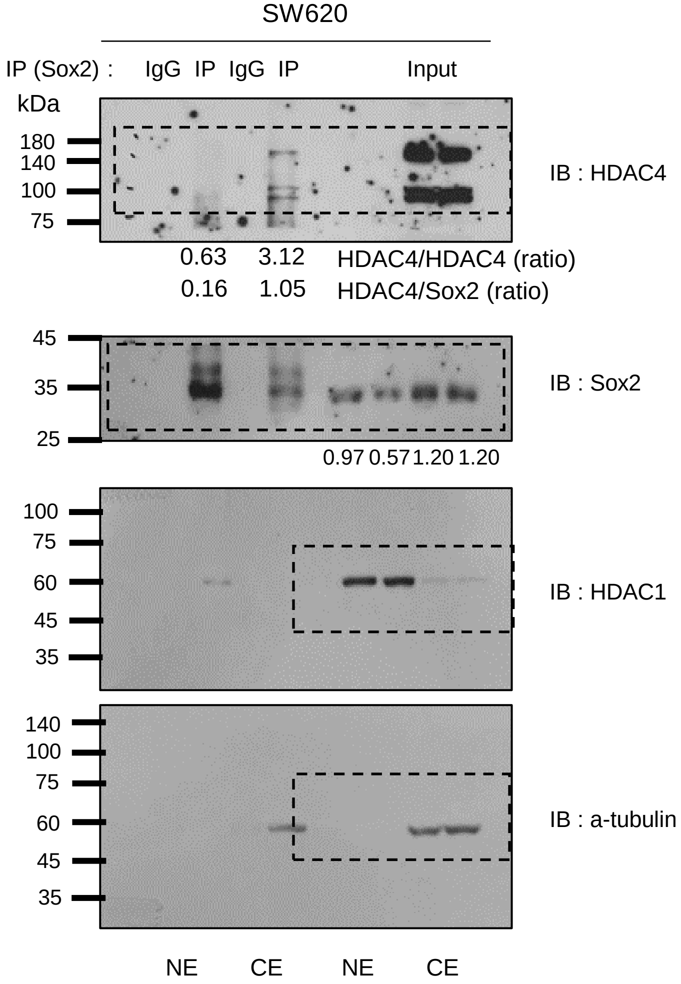

Supplementary Figure S7.

(Figure 5 H)

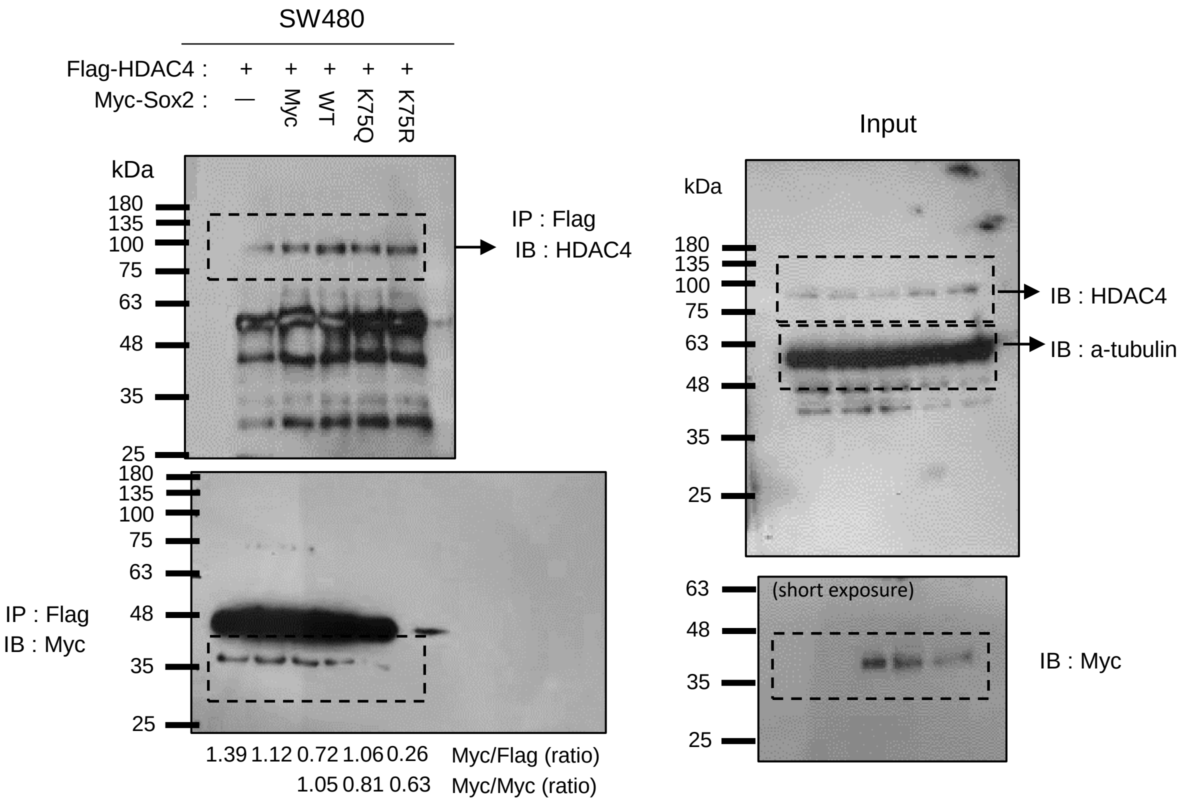

(Figure 6 C)

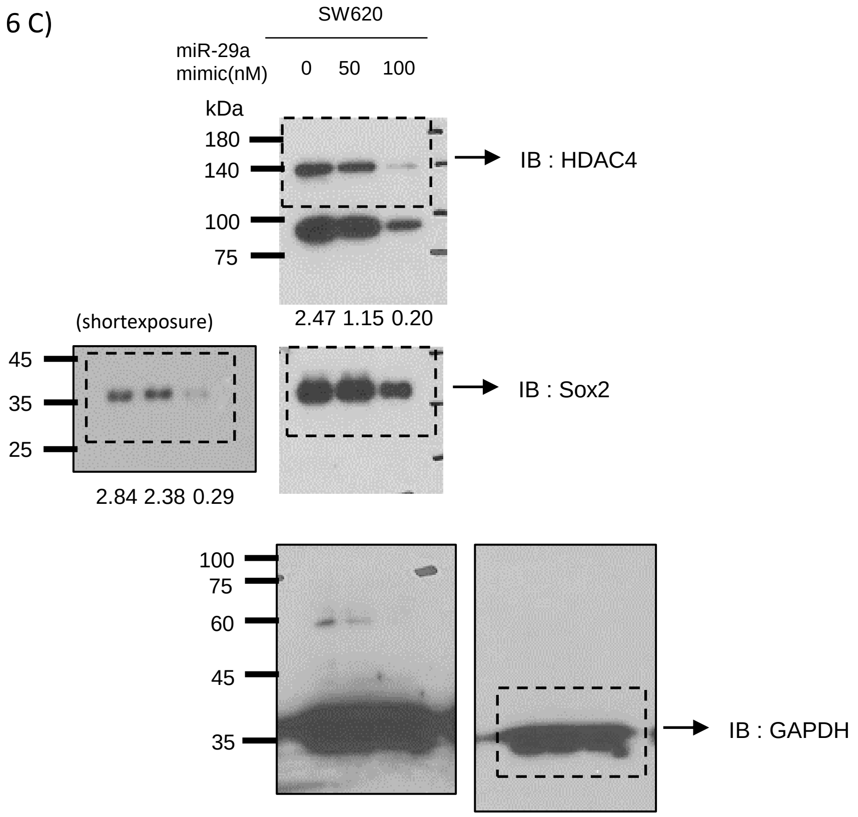

Supplementary Figure S7.

(Figure 7 B)

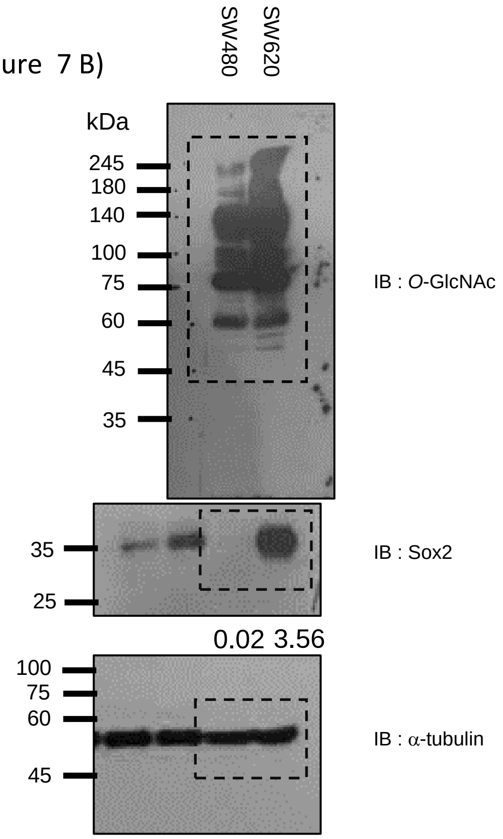

(Figure 7 D)

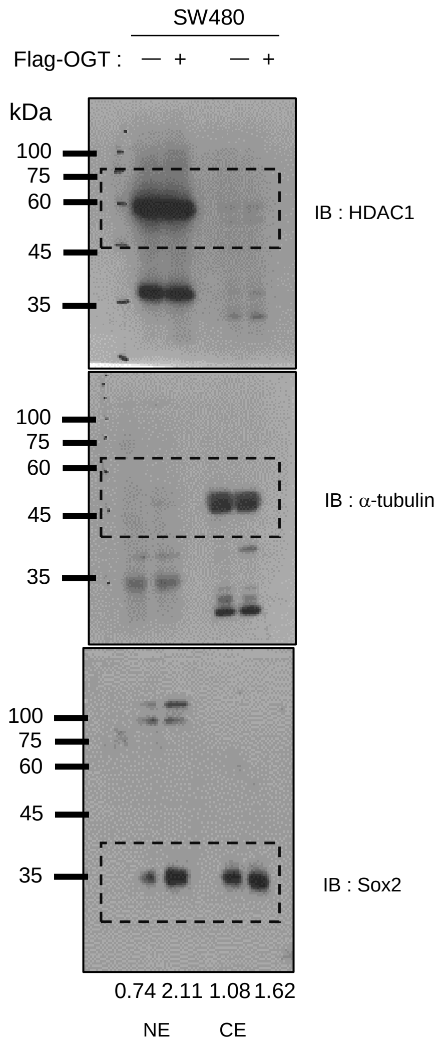

(Figure 7 F)

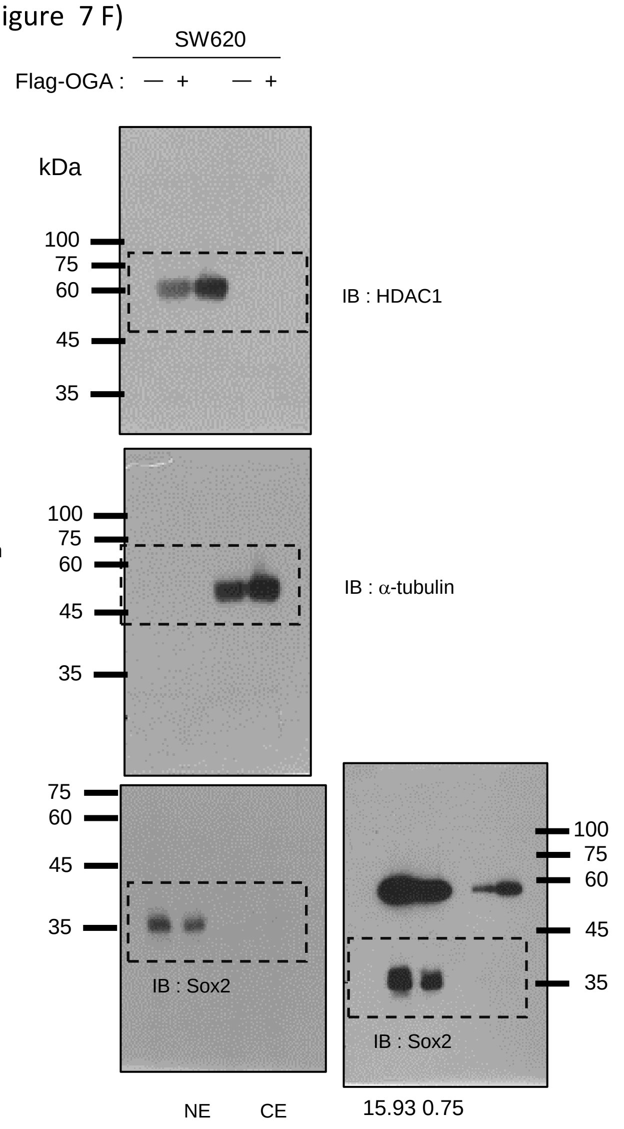

Supplementary Figure S7.

(Figure 7 G)

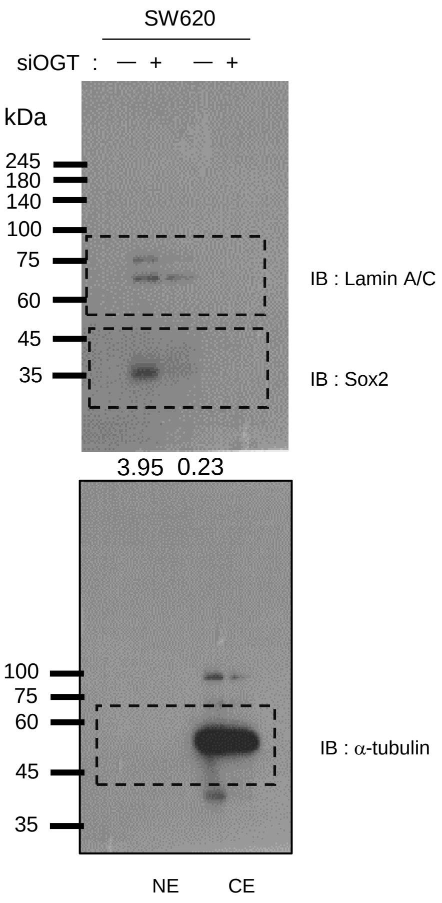

(Sub Figure 3 C)

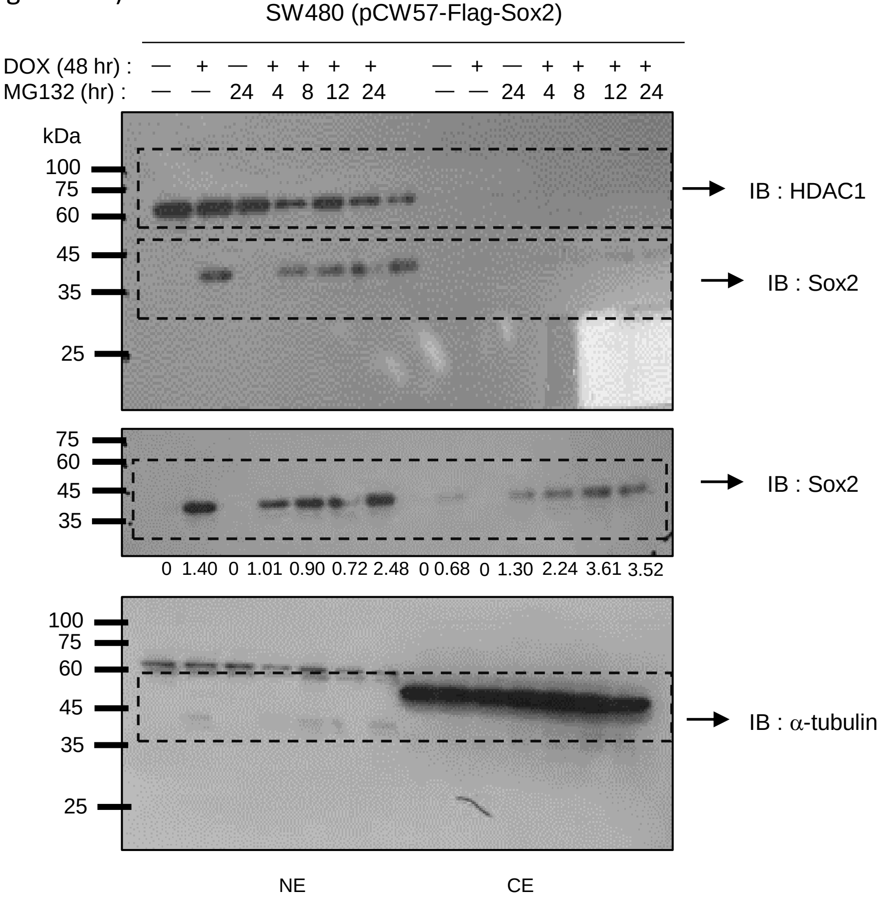

Supplementary Figure S7.

(Sub Figure 5 A)

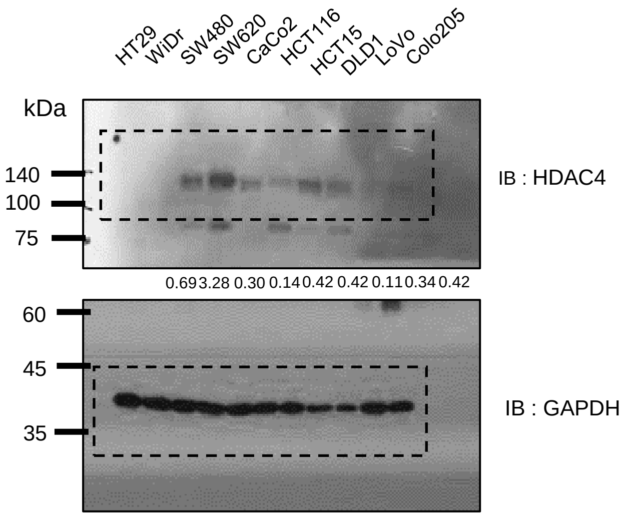

(Sub Figure 5 D)

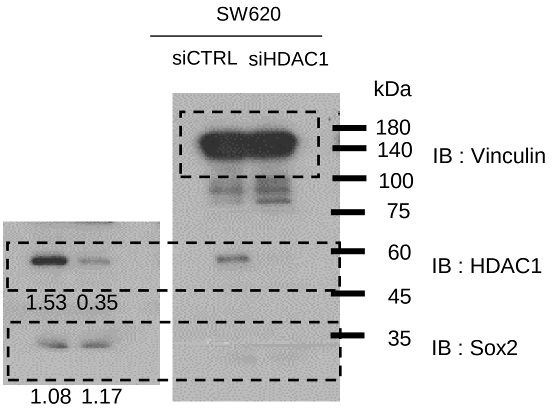

(Sub Figure 5 E)

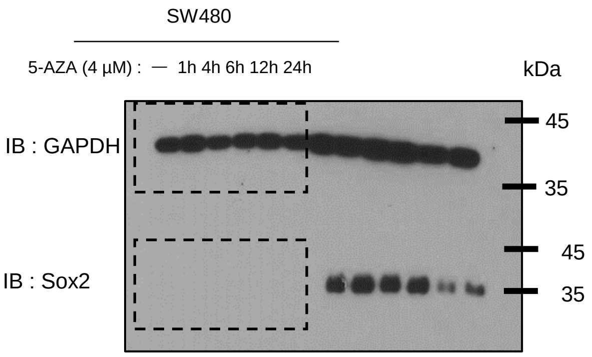

Supplement: Supplementary file 1 [file cancers-16-01035-s001.zip › cancers-2875833-supplementary.pdf]
