# Supplementary material for: A Comprehensive Understanding of Post-Translational Modification of Sox2 via Acetylation and O-GlcNAcylation in Colorectal Cancer
Source: Cancers (Basel). 2024 Mar 3;16(5):1035. doi: 10.3390/cancers16051035 (PMC10931190; doi:10.3390/cancers16051035)
Supplement: Supplementary file 1 [file cancers-16-01035-s001.zip › cancers-2875833-Table S1.pdf]

Table S1. Comprehensive list of antibodies, biological samples, agents, cell lines, and oligonucleotides used.

REAGENT or RESOURCE	SOURCE	IDENTIFIER
Antibodies (concentrations used)		
anti-SoX2 (1:1000)	Cell Signaling Technology	Cat. #2748S; RRID:AB_823640
anti-HDAC4 (1:1000)	Cell Signaling Technology	Cat. #2072; RRID:AB_2232915
anti-HDAC4 (1:1000)	Santa Cruz Biotechnology	Cat. #sc-46672, RRID:AB_627706
anti-HDAC1 (1:1000)	Santa Cruz Biotechnology	Cat. #sc-81598; RRID:AB_2118083
anti-p300 (NM11) (1:1000)	Santa Cruz Biotechnology	Cat. #sc-32244; RRID:AB_2628076
anti-p300 (F4) (1:1000)	Santa Cruz Biotechnology	Cat. #sc-48343; RRID:AB_628075
anti-Ac-lysine (AKL5C1) (1:1000)	Santa Cruz Biotechnology	Cat. #sc-32268; RRID:AB_627898
anti-Acetylated-lysine (1:1000)	Cell Signaling Technology	Cat. #9441S; RRID:AB_331805
Anti-OGT (D1D8Q) (1:1000)	Cell Signaling Technology	Cat. #24083S; RRID:AB_2716710
Anti-O-GlcNAc (CTD110.6) (1:1000)	Biolegend	Cat. #838004; RRID:AB_2629520
Anti-CRM1 (C-1) (1:1000)	Santa Cruz Biotechnology	Cat. #sc-74454; RRID:AB_1122704
Anti-c-Myc (1:1000)	Santa Cruz Biotechnology	Cat. #sc-40; RRID:AB_2857941
Anti-Flag (DYKDDDDK Tag) (1:1000)	Cell Signaling Technology	Cat. #14793; RRID:AB_2572291
Anti-α-tubulin (1:5000)	Sigma-Aldrich	Cat. #T9026; RRID:AB_477593
Anti-Lamin A/C (346) (1:4000)	Santa Cruz Biotechnology	Cat. #sc-7293; RRID:AB_627874
Anti-GAPDH (1:5000)	Abfrontier	Cat. #LF-PA0018;
Anti-Vinculin (7F9) (1:5000)	Santa Cruz Biotechnology	Cat. #sc-73614; RRID:AB_1131294
Goat anti-rabbit secondary antibody, HRP (1:5000)	Thermo Fisher Scientific	Cat. #32260, RRID:AB_1965959
Goat anti-mouse secondary antibody, HRP (1:5000)	Thermo Fisher Scientific	Cat. #32230, RRID:AB_1965958
FITC-Conjugated Affinity Purified anti-Mouse IgG (1:5000)	ABM	Cat. #SF023
FITC-Conjugated Affinity Purified anti-Rabbit IgG (1:5000)	ABM	Cat. #SF025
Cy3-Conjugated Affinity Purified anti-Mosue IgG (1:5000)	ABM	Cat. #SF023
Cy3-Conjugated Affinity Purified anti-Rabbit IgG (1:5000)	ABM	Cat. #SF024
Bacterial and virus strains		
Stellar™ Competent Cells	Clontech	Cat. #636763

E. coli DH5α Competent Cells	Takara	Cat. #9057
Biological samples		
CRC Patient-Derived Organoids (PDOs)	This paper	N/A
RNA Sequencing of primary CRC tumors	This paper	N/A
Tissue of primary CRC tumors	This paper	N/A
Chemicals, peptides, and recombinant proteins		
High glucose DMEM	Cytiva	Cat. #SH30022.01
FBS	Thermo Fisher Scientific	Cat. #16000044
Advanced DMEM/F-12	Thermo Fisher Scientific	Cat. #12634028
L-glutamine	Thermo Fisher Scientific	Cat. #25030024
MEM Non-Essential Amino Acids	Thermo Fisher Scientific	Cat. #11140050
GlutaMAX™ Supplement	Thermo Fisher Scientific	Cat. #35050061
Trypsin	Cytiva	Cat. #sh30042.02
Penicillin and streptomycin (P/S)	Lonza	Cat. #17–602E
Protease inhibitor	Roche	Cat. #11836-153-001
TRIzol™ Reagent	Thermo Fisher Scientific	Cat. #15596018
5x RT premix Mix	HK Genomics	Cat. #73201
TOPreal™ qPCR 2X PreMIX (SYBR Green)	Enzynomics	Cat. #RT501M
M-MLV Reverse Transcriptase, RNaseH^{+}	ELPIS Biotech	Cat. #EBP-1028
QIAzol lysis reagent	Qiagen	Cat. #79306
Chloroform	Sigma-Aldrich	Cat. #25666
Isopropanol	Sigma-Aldrich	Cat. #563935
VY-3-153 (ACSS2 inhibitor)	Aobious	Cat. #AOB12492
Trichostatin A (HDAC inhibitor)	Sigma-Aldrich	Cat. #T8552
5-azacytidine (DNA Methyltransferase Inhibitor)	Sigma-Aldrich	Cat. # 260920
MG-132 (Proteasome inhibitor)	Sigma-Aldrich	Cat. #M7449
C646 (Histone Acetyltransferase p300 inhibitor)	Sigma-Aldrich	Cat. #SML0002
Thiamet-G (*O*-GlcNAcase inhibitor)	Sigma-Aldrich	Cat. #SML0244
Polybrene	Sigma-Aldrich	Cat. #107689
Doxycycline	Sigma-Aldrich	Cat. #D9891

TrypLE™ Express Enzyme	Thermo Fisher Scientific	Cat. #12605028
Protease inhibitor	Roche	Cat. #11836-153-001
Sodium Dodecyl Sulfate (SDS)	Sigma-Aldrich	Cat.# 11667289001
Nonidet™ P 40 Substitute	Sigma-Aldrich	Cat.#74385
Triton-X100	Sigma-Aldrich	Cat.# T8787
Tween-20	Sigma-Aldrich	Cat. #P2287
Albumin	GenDEPOT	Cat. #A0100-010
Y-27632 (ROCK inhibitor)	Santa Cruz Biotechnology	Cat. #sc-281642A
Matrigel matrix	Corning	Cat. #354234
human EGF Recombinant Protein	R&D	Cat. #236-EG
CHIR99021	Sigma-Aldrich	Cat. #SML1046
Gastrin	Sigma-Aldrich	Cat. #G9145
SB202190	Sigma-Aldrich	Cat. #S7067
A8301	Sigma-Aldrich	Cat. #SML0788
N-Acetyl-L-cysteine (NAC)	Sigma-Aldrich	Cat. #A9165
Nicotinamide	Sigma-Aldrich	Cat. #N3376
rhNoggin	R&D	Cat. #6057-NG
N2	Thermo Fisher Scientific	Cat. #17502048
B27	Thermo Fisher Scientific	Cat. #17504044
jetPRIME® *in vitro* DNA transfection reagent	Polyplus	Cat. #101000046
INTERFEin	Polyplus	Cat. #101000028
Skim milk	BD Difco	Cat. #232100
LB Broth, Miller	Sigma-Aldrich	Cat. #L3522
Agar, Bacto	GenDEPOT	Cat. #A2007-050
4,6-diamidino-2-phenylindole (DAPI)	Sigma-Aldrich	Cat. #D9542
Sodium citrate tribasic dihydrate	Sigma-Aldrich	Cat. #S4641
Hydrogen Peroxide	Duksan	Cat. #7722-84-1
Xylene Substitute	Sigma-Aldrich	Cat. #A5597-1GAL
Critical commercial assays		
H&E staining kit (Hematoxylin and Eosin)	Abcam	Cat. #ab245880

miRCURY LNA miRNA PCR Starter Kit	Qiagen	Cat. #3390320
Deposited data		
RNA sequencing raw and analyzed data	This paper	
RNA sequencing raw data	This paper	GEO repository (GSE)
LC-MS/MS	This paper	
mRNA array	This paper	
miRNA array	This paper	
Experimental models: Cell lines		
SW480	ATCC	Cat. #CCL-228, RRID:CVCL_0546
SW620	ATCC	Cat. #CCL-227, RRID:CVCL_0547
HT-29	ATCC	Cat. #HTB-38, RRID:CVCL_0320
WiDr	ATCC	Cat. #CCL-218, RRID:CVCL_2760
Caco2	ATCC	Cat. #HTB-37, RRID:CVCL_0025
RKO	ATCC	Cat. #CRL-2577, RRID:CVCL_0504
HCT116	ATCC	Cat. #CCL-247, RRID:CVCL_0291
HCT15	ATCC	Cat. #CCL-225, RRID:CVCL_0292
Colo205	ATCC	Cat. #CCL-222, RRID:CVCL_0218
LoVo	ATCC	Cat. #CCL-229, RRID:CVCL_0399
DLD-1	ATCC	Cat. #CCL-221, RRID:CVCL_0248
L-Wnt 3a	ATCC	Cat.# CRL-2647 RRID:CVCL_0635
L-WRN	ATCC	Cat.#CRL-3276, RRID:CVCL_DA06
CRC Patient-Derived Organoids (PDOs)	This paper	N/A
Oligonucleotides		
Sox2 F-CAAGATGCACAACTTGGAGA	Bionics	N/A
Sox2 R- GCTTAGCCTCGTCGATGAAC	Bionics	N/A
HDAC4 F-GGTTTATTCTGATTGAGAACTGG	Bionics	N/A
HDAC4 R-ATTGTAAACACAAGTGCTCGC	Bionics	N/A
HDAC1 F-GGAAATCTATCGCCCTCACA	Bionics	N/A
HDAC1 R-AACAGGCCATCGAATACTGG	Bionics	N/A
OGT F-CTGTCACCCTTGACCCAAAT	Bionics	N/A

OGT R-ACGAAGATAAGCTGCCACAG	Bionics	N/A
OGA F-TGGAAGACCTTGGGTTATGG	Bionics	N/A
OGA R-TGCTCAGCTTCTTCCACTGA	Bionics	N/A
GAPDH F-TGGACCTGACCTGCCGTCTA	Bionics	N/A
GAPDH R-CCCTGTTGCTGTAGCCAAATTC	Bionics	N/A
HDAC1 siRNA	GE Dharmacon	Cat.#M-003493-02
HDAC4 siRNA	GE Dharmacon	Cat.#M-003497-03
OGT siRNA	Seo et al. [45]	N/A
OGA siRNA	Seo et al. [45]	N/A
miR29a primer	Applied Biosystems	Cat.#002112
miRNA positive control	GE Dharmacon	Cat.#CP-004500-01-05
miRNA negative control	GE Dharmacon	Cat.#CN-001000-04-05
Recombinant DNA		
pCDNA Mock	Yoon et al. [29]	N/A
pCDNA-Sox2 WT	Yoon et al. [29]	N/A
pCDH-Myc	This paper	N/A
pCDH-Myc-Sox2 WT	This paper	N/A
pCDH-Myc-Sox2 K75A	This paper	N/A
pCDH-Myc-Sox2 K75Q	This paper	N/A
pCDH-Myc-Sox2 K75R	This paper	N/A
pCDH-Myc-Sox2 K75A/S246A	This paper	N/A
pCDH-Myc-Sox2 K75Q/S246A	This paper	N/A
pCW57-FLAG	This paper	N/A
pCW57-FLAG-Sox2 WT	This paper	N/A
p3XFlag-CMV™-7.1-OGT	Seo et al. [45]	N/A
p3XFlag-CMV™-7.1-OGA	Seo et al. [45]	N/A
Software and algorithms		
GraphPad Prism 7 Software	GraphPad Software	N/A
ImageJ and Fiji	https://imagej.net/	N/A
Open Comet v1.3.1	www.cometbio.org	N/A

GSEA (Gene Set Enrichment Analysis)	https://www.gsea-msigdb.org/gsea/index.jsp	N/A
DESeq2	(Love et al., 2014)	N/A
R v3.2.1	https://www.r-project.org/	N/A
